# Supplementary figures and images for: Endogenous oligomer formation underlies DVL2 condensates and promotes Wnt/β-catenin signaling
Source: eLife. 2024 Dec 9;13:RP96841. doi: 10.7554/eLife.96841 (PMC11627551; doi:10.7554/eLife.96841)

To Panel A

unedited

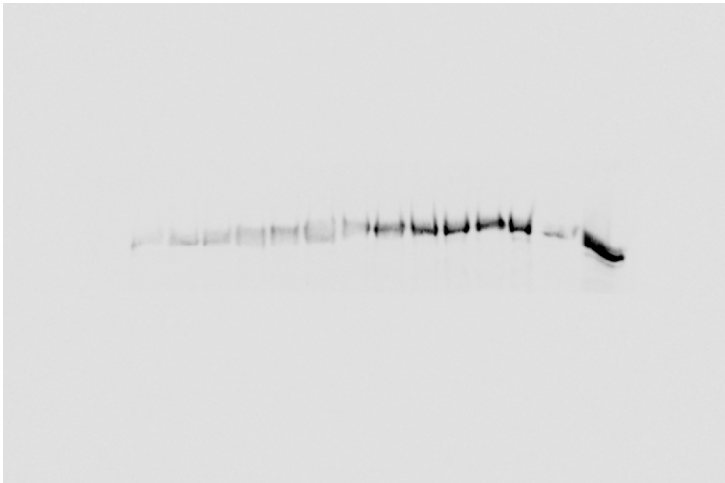

labelled

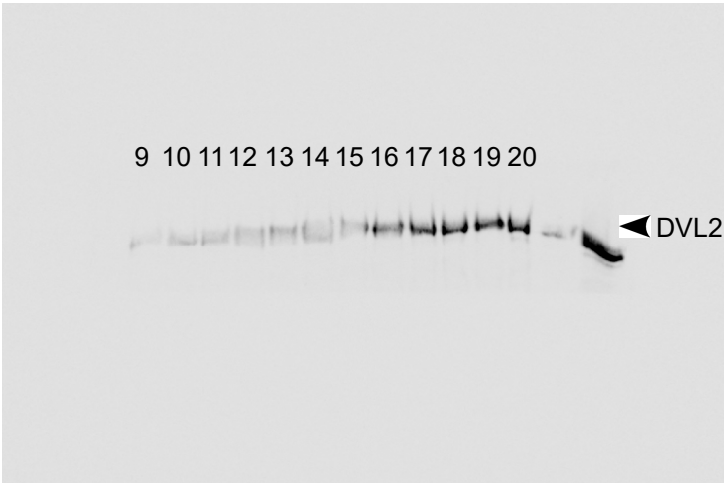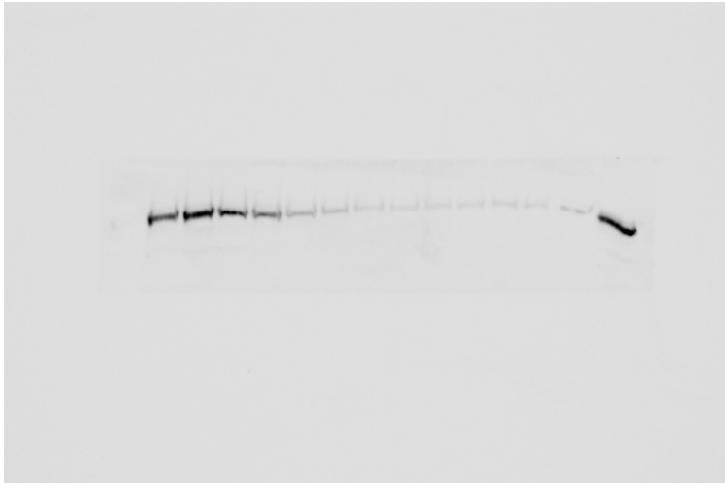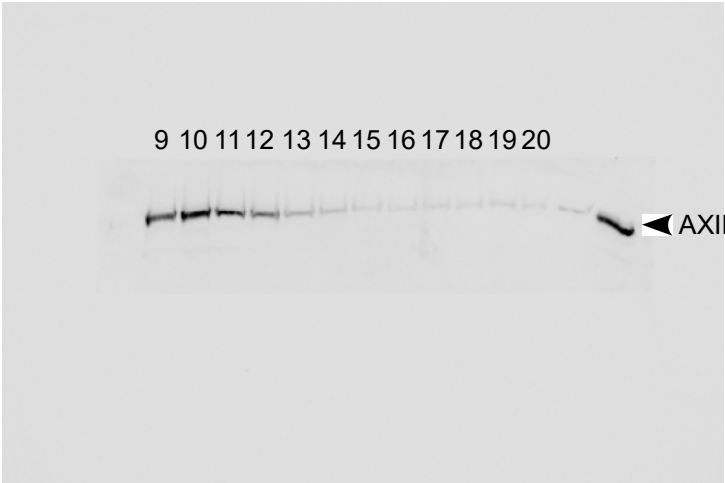

Supplement: Figure 1—source data 2. [file elife-96841-fig1-data2.zip › Figure 1-source data 2/Figure 1A.pdf]

To Panel B

unedited

labelled

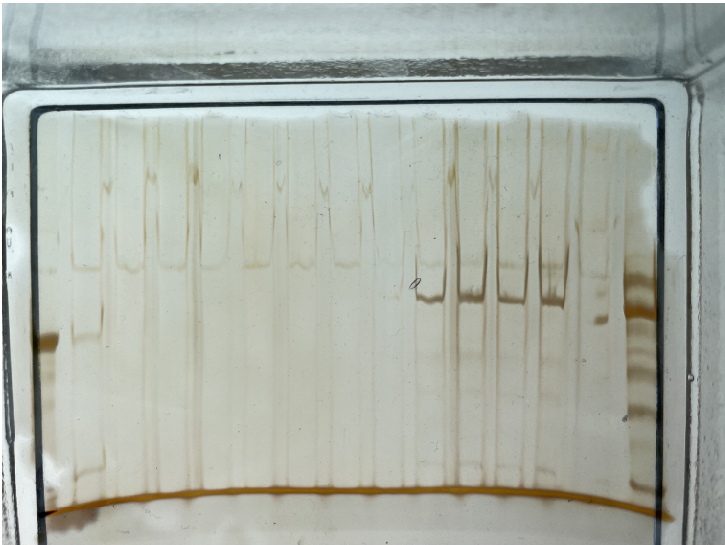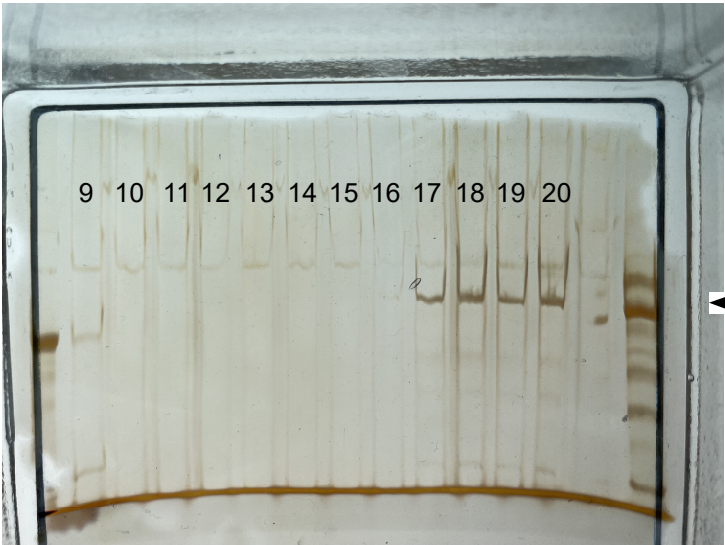

9 10 11 12 13 14 15 16 17 18 19 20

◀ TG

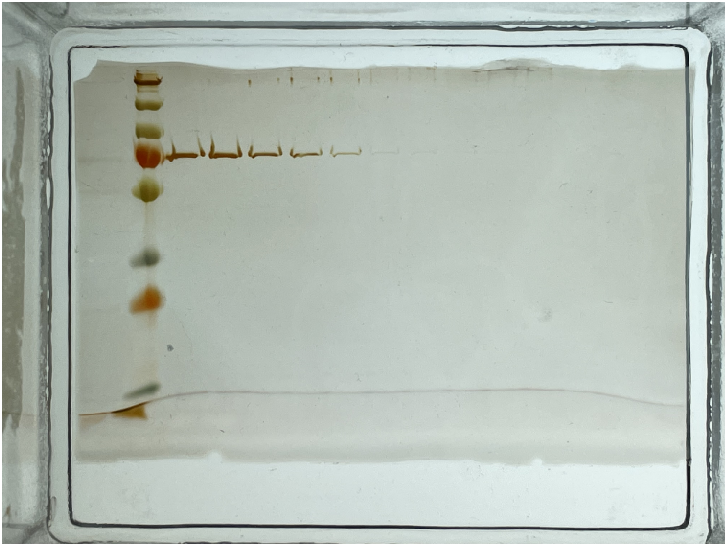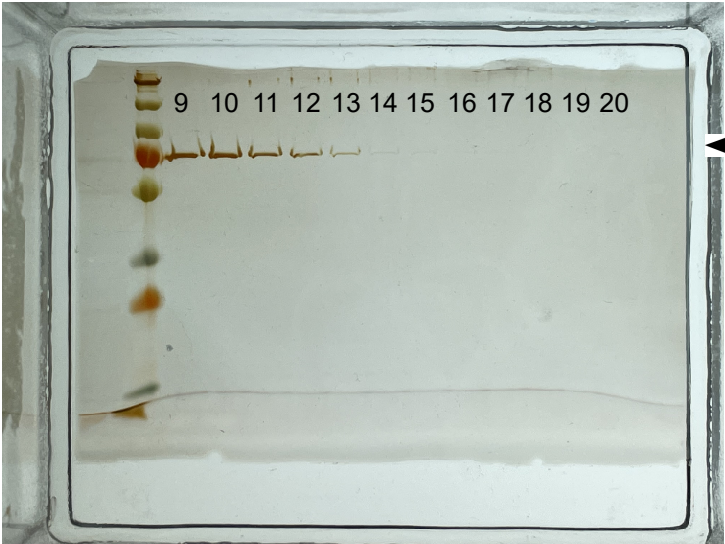

9 10 11 12 13 14 15 16 17 18 19 20

◀ ALB

Supplement: Figure 1—source data 2. [file elife-96841-fig1-data2.zip › Figure 1-source data 2/Figure 1B.pdf]

To Panel C

unedited

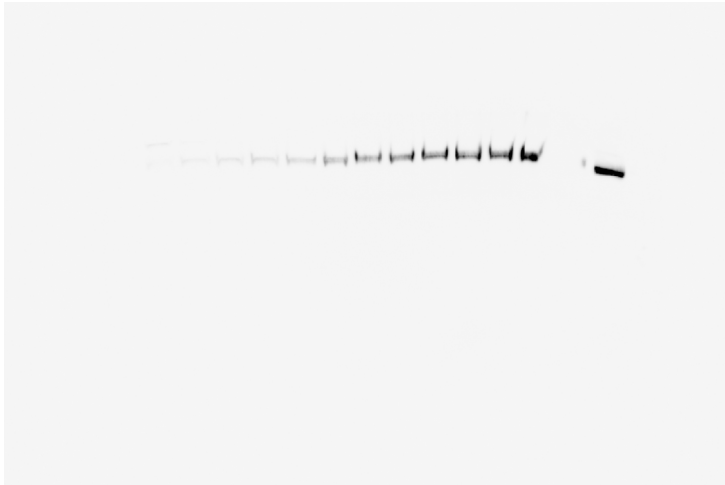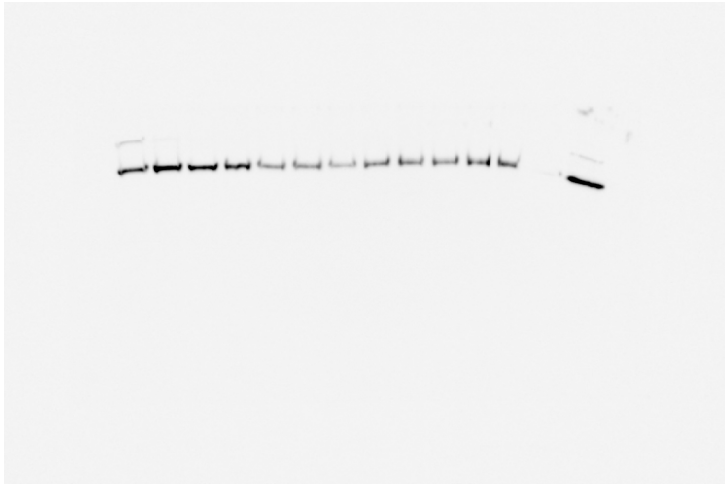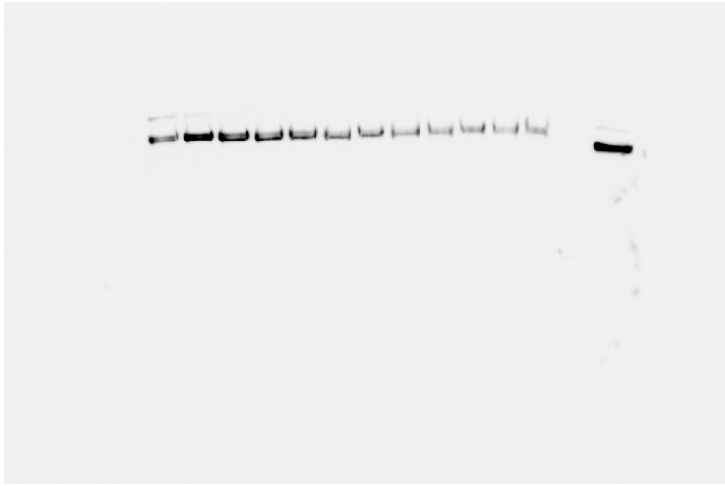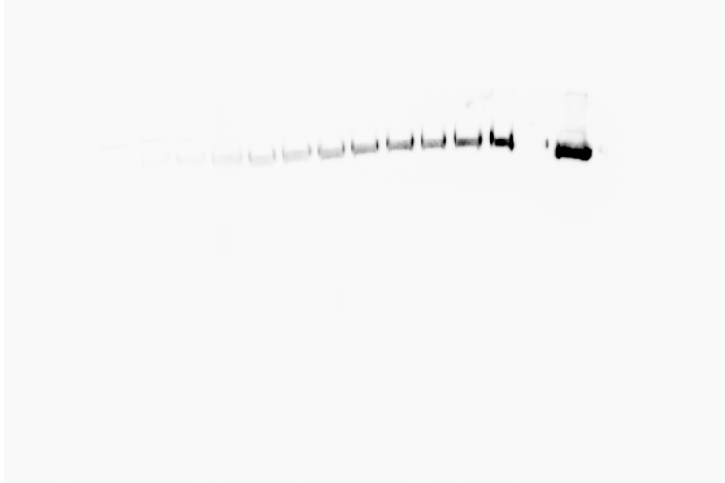

labelled

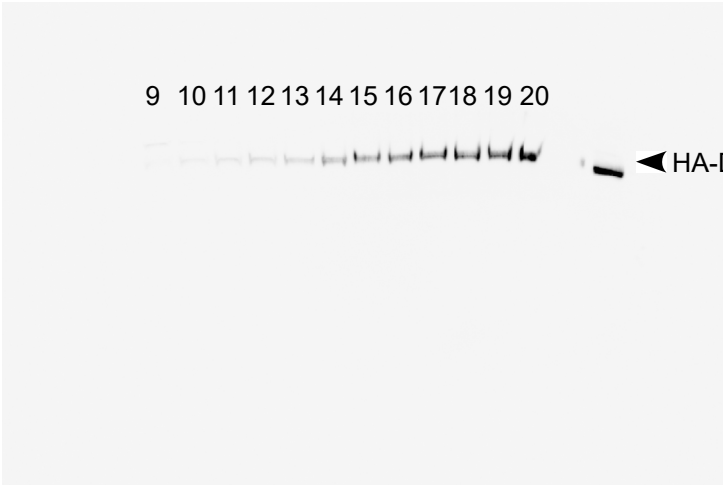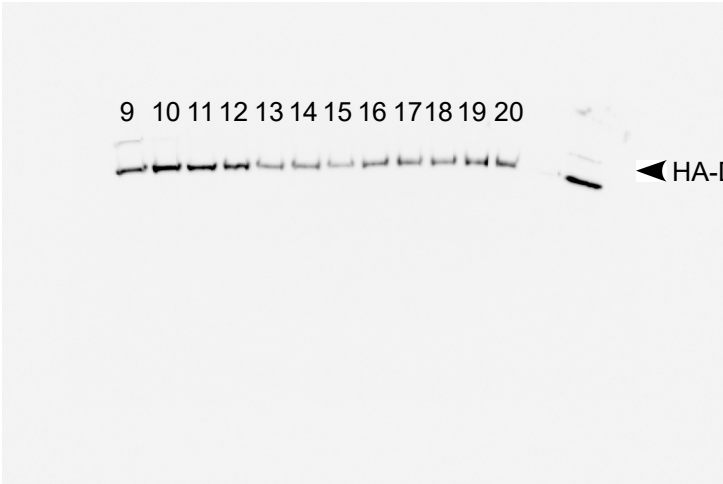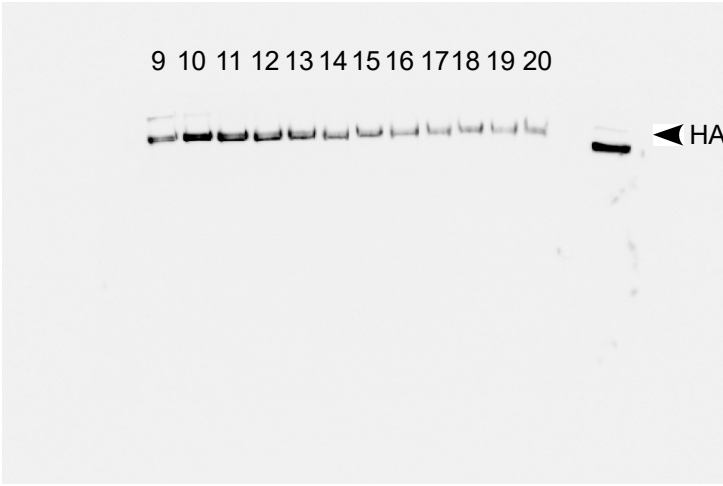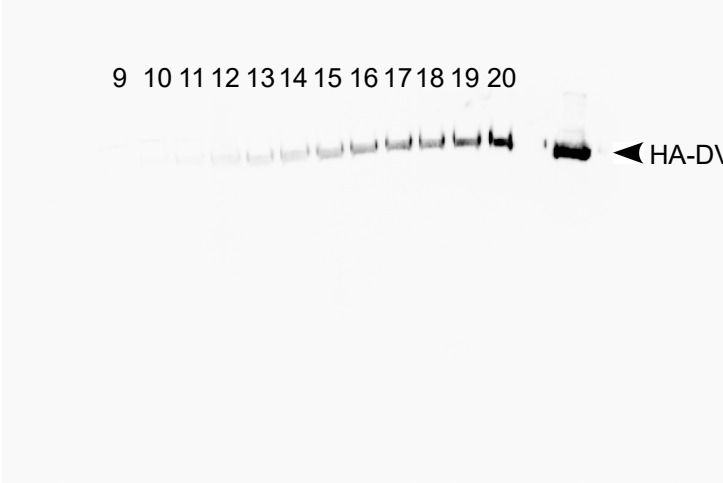

Supplement: Figure 1—source data 2. [file elife-96841-fig1-data2.zip › Figure 1-source data 2/Figure 1C.pdf]

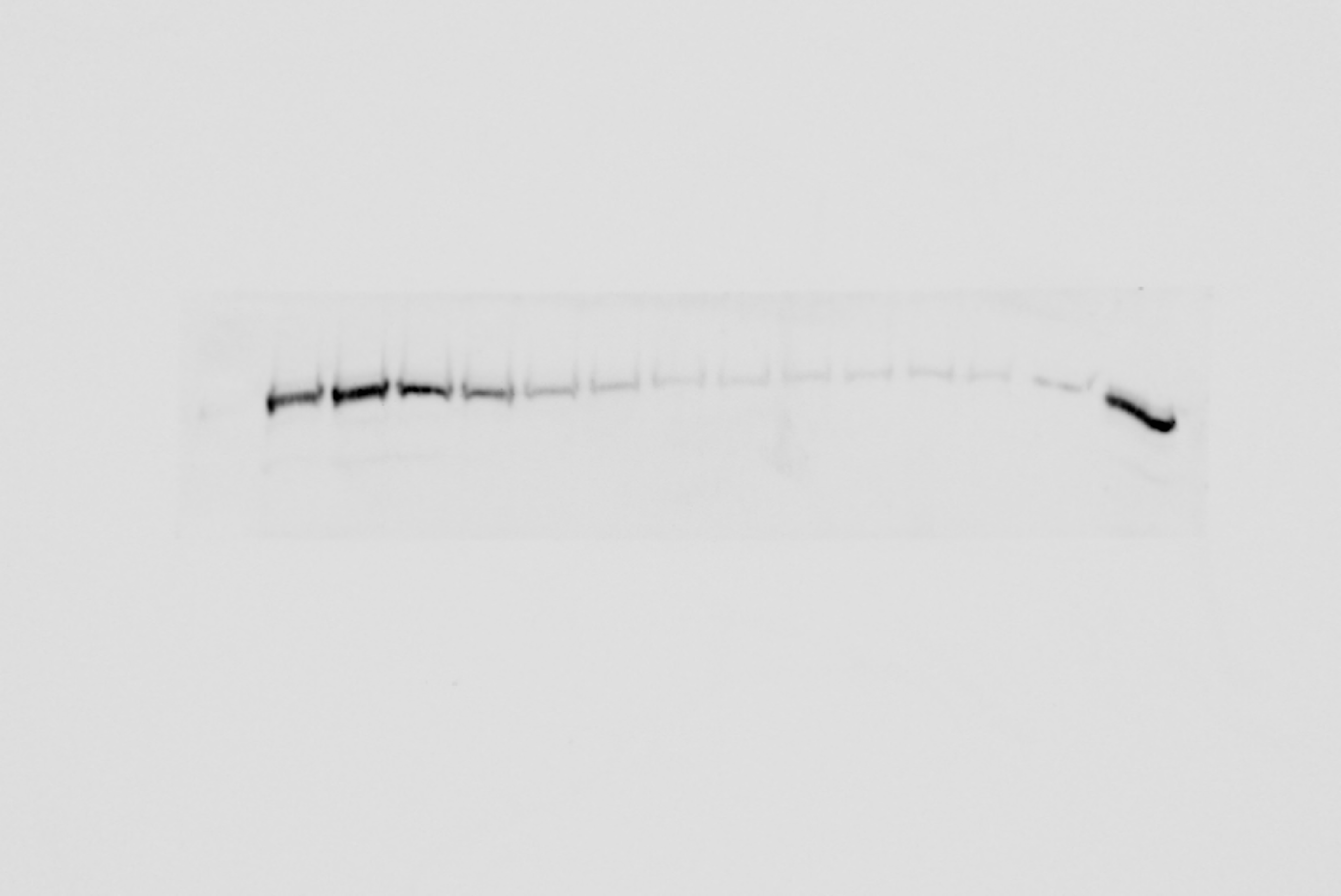

Supplement: Figure 1—source data 3. [file elife-96841-fig1-data3.zip › Figure 1-source data 3/Figure 1A - AXIN1.tif]

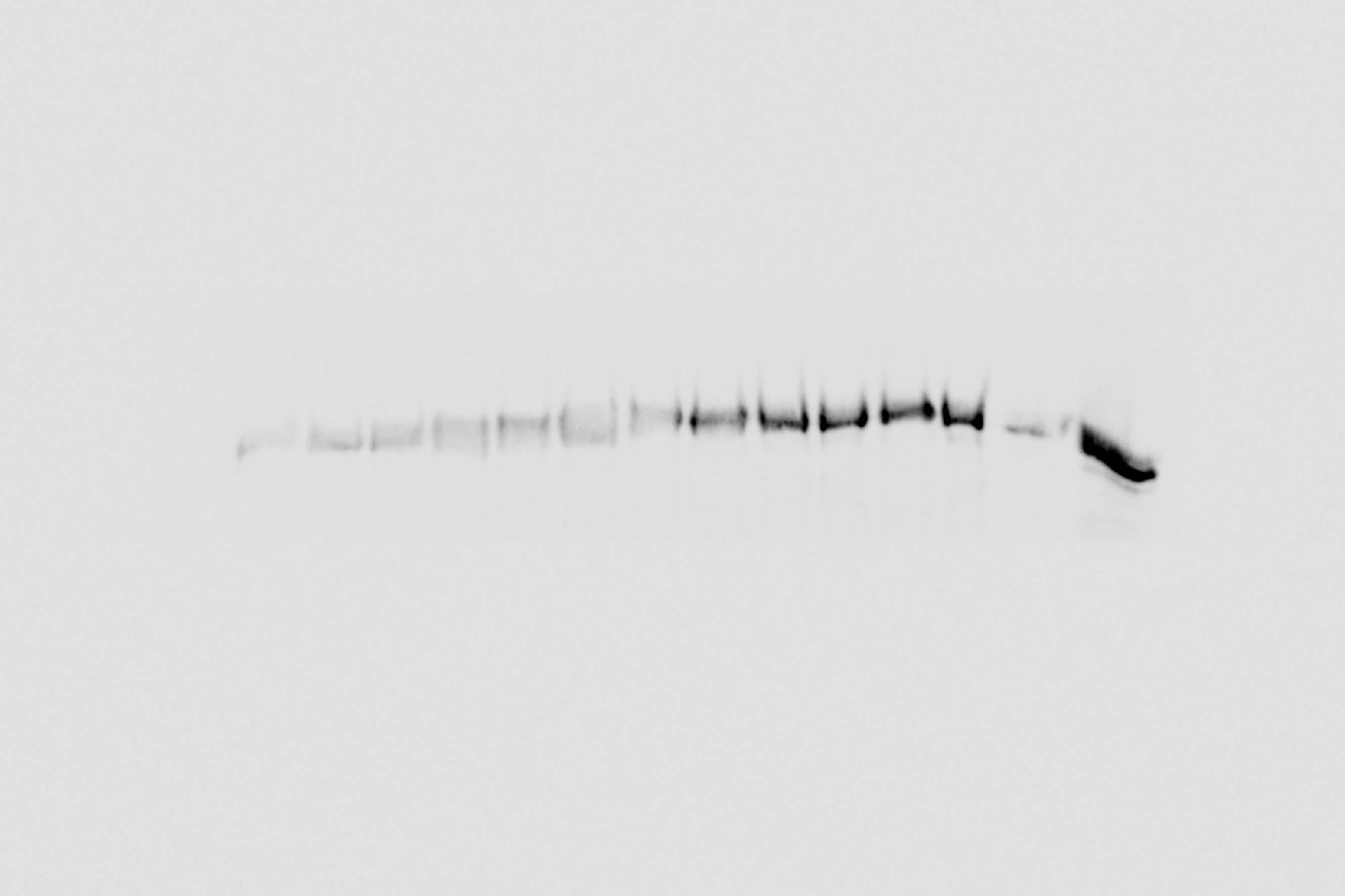

Supplement: Figure 1—source data 3. [file elife-96841-fig1-data3.zip › Figure 1-source data 3/Figure 1A - DVL2.tif]

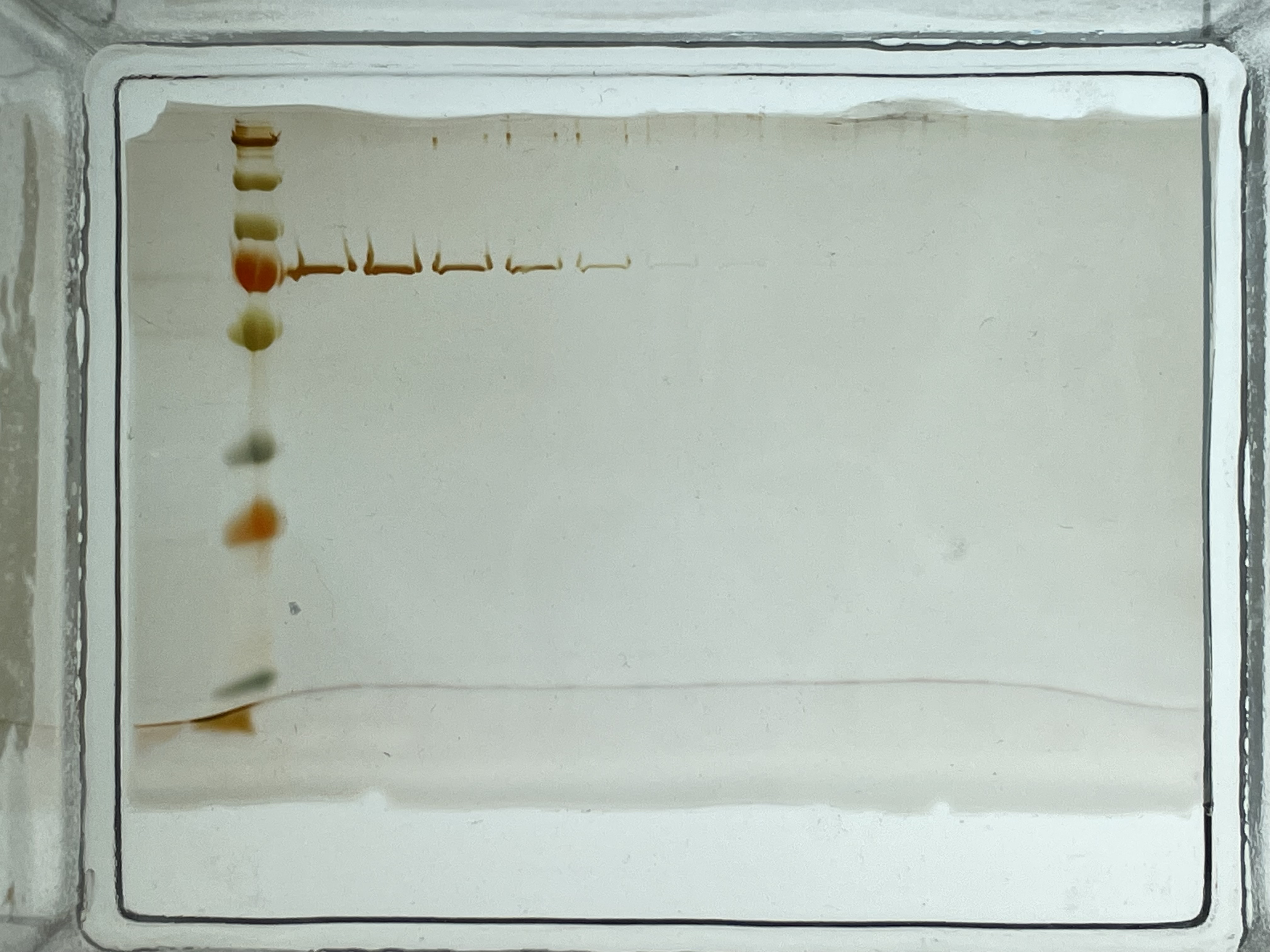

Supplement: Figure 1—source data 3. [file elife-96841-fig1-data3.zip › Figure 1-source data 3/Figure 1B - ALB.tif]

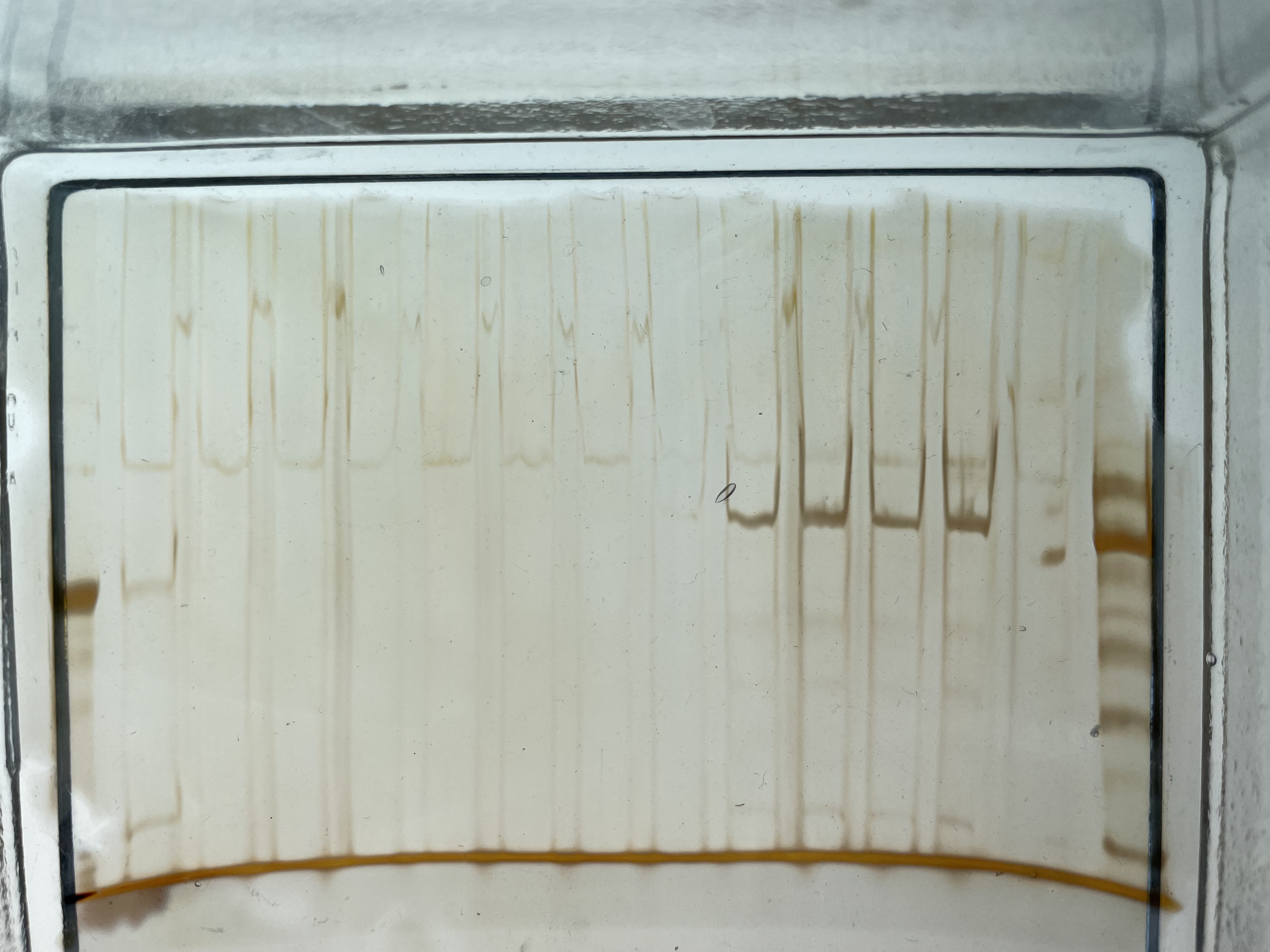

Supplement: Figure 1—source data 3. [file elife-96841-fig1-data3.zip › Figure 1-source data 3/Figure 1B - TG.tif]

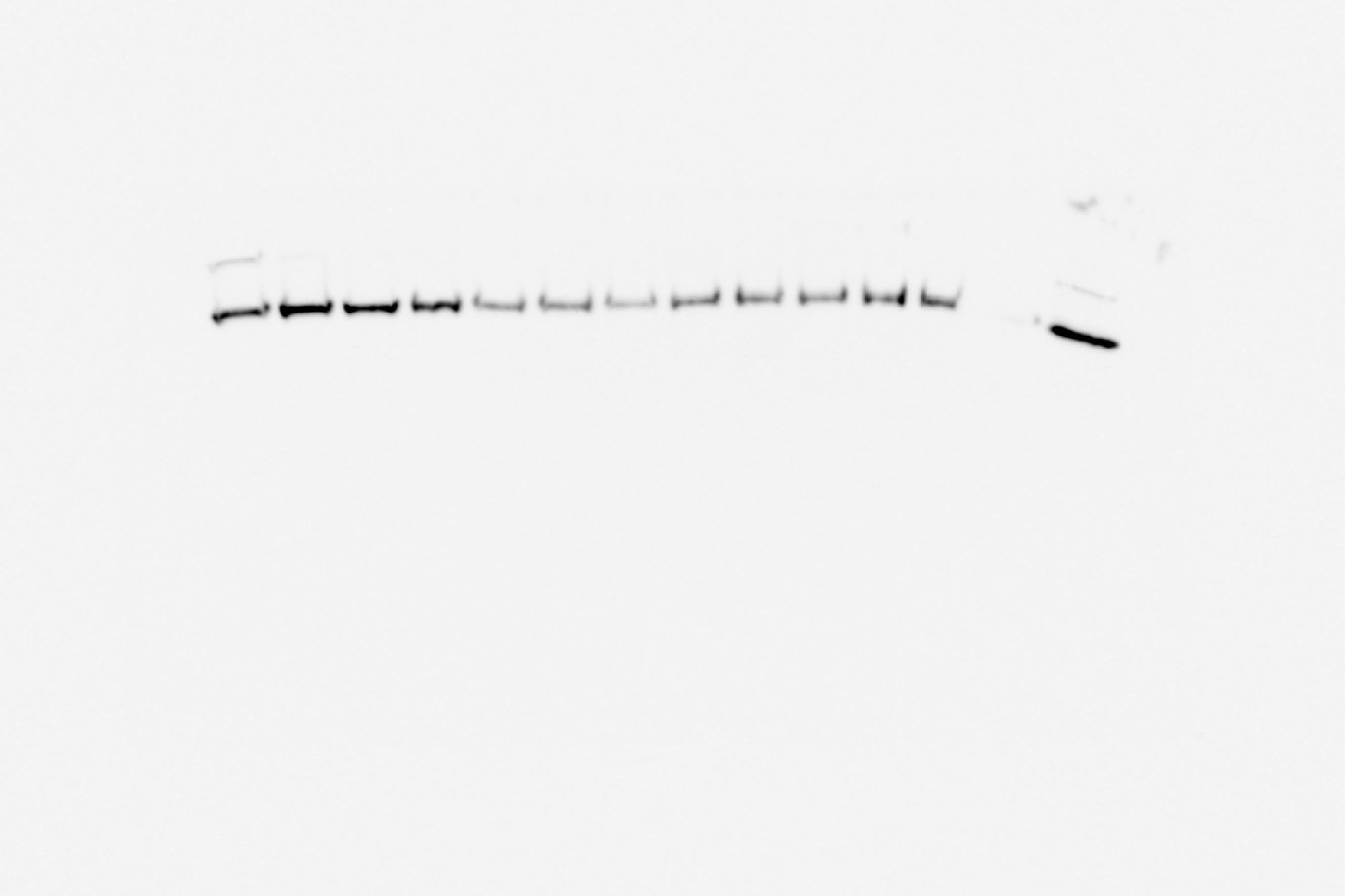

Supplement: Figure 1—source data 3. [file elife-96841-fig1-data3.zip › Figure 1-source data 3/Figure 1C - DVL1.tif]

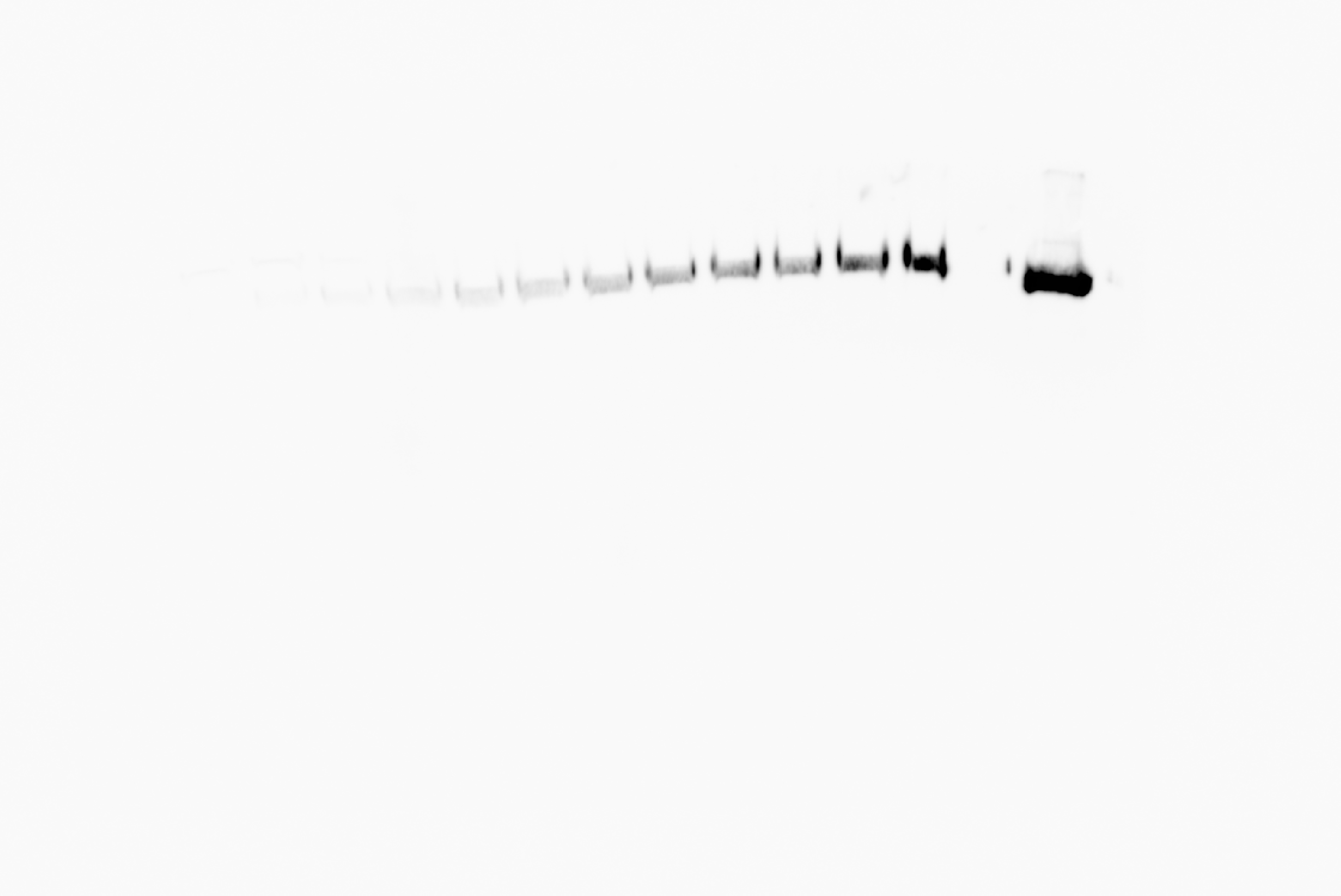

Supplement: Figure 1—source data 3. [file elife-96841-fig1-data3.zip › Figure 1-source data 3/Figure 1C - DVL2 M2.tif]

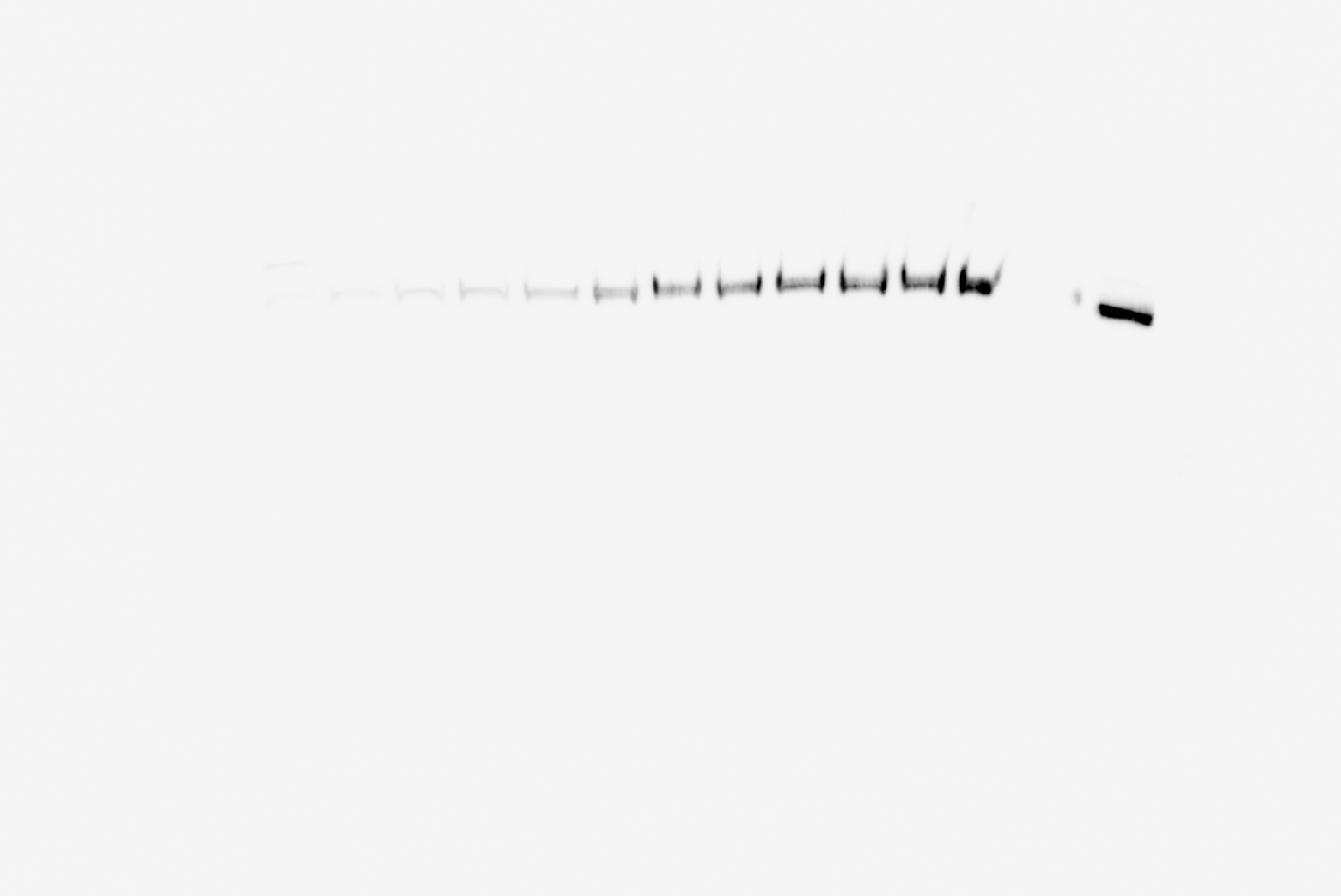

Supplement: Figure 1—source data 3. [file elife-96841-fig1-data3.zip › Figure 1-source data 3/Figure 1C - DVL2.tif]

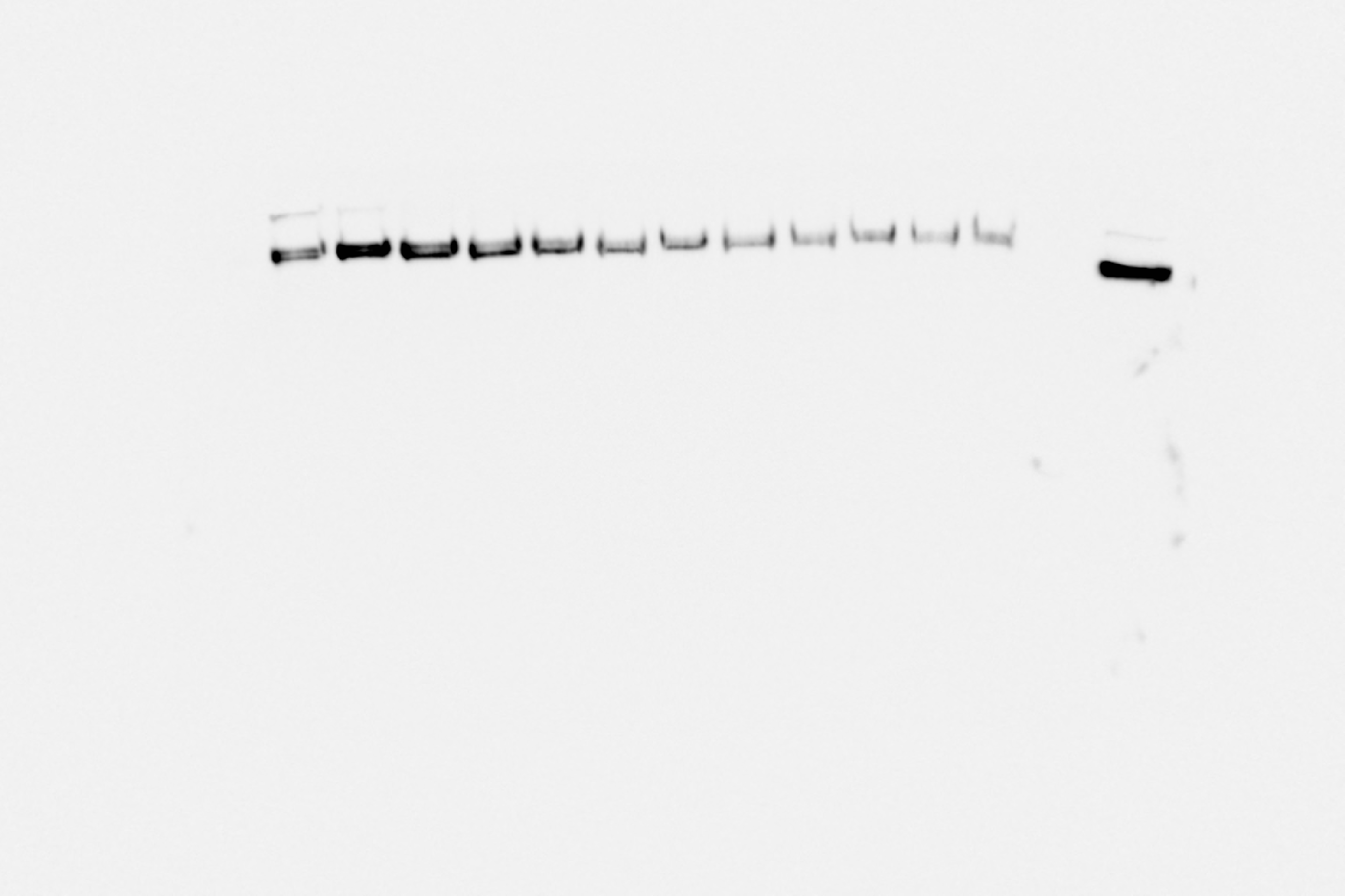

Supplement: Figure 1—source data 3. [file elife-96841-fig1-data3.zip › Figure 1-source data 3/Figure 1C - DVL3.tif]

To Panel A

unedited

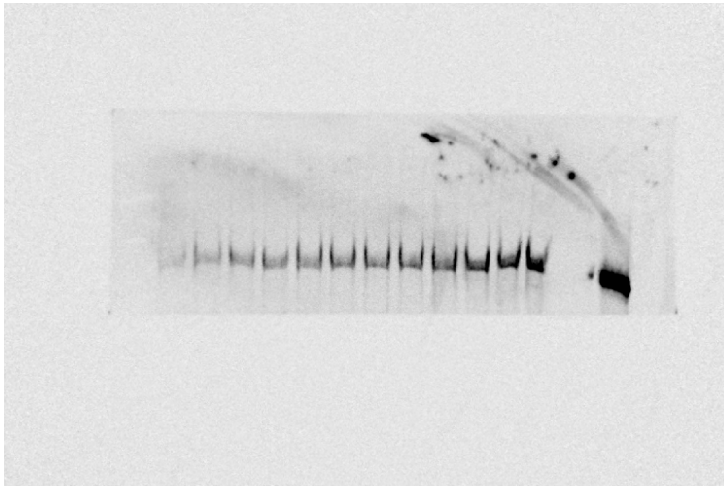

labelled

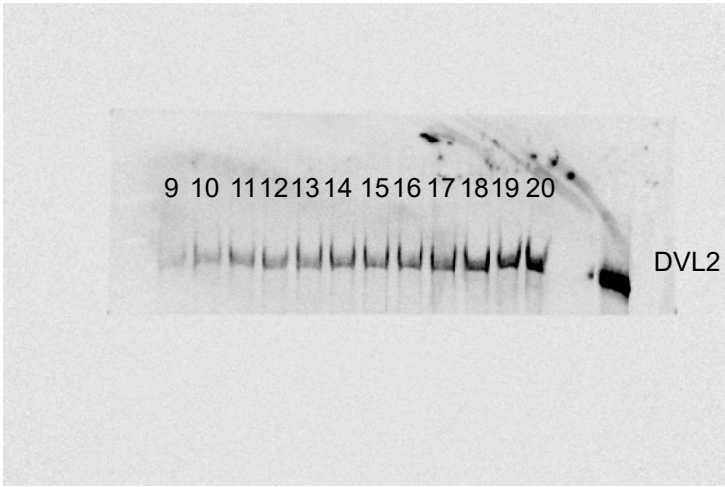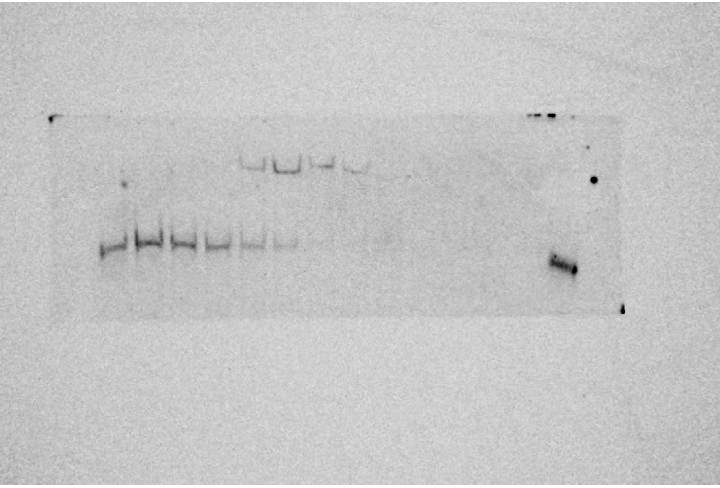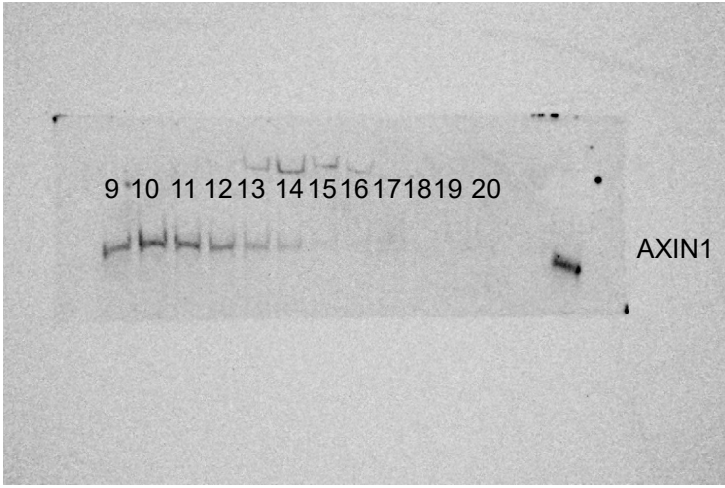

To Panel B

unedited

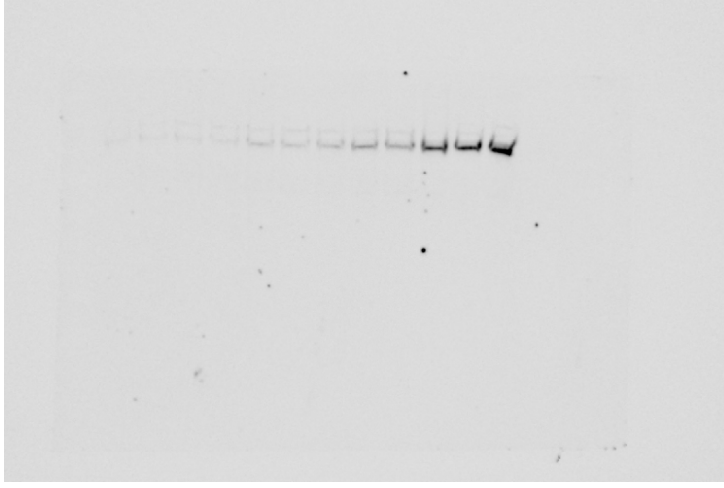

labelled

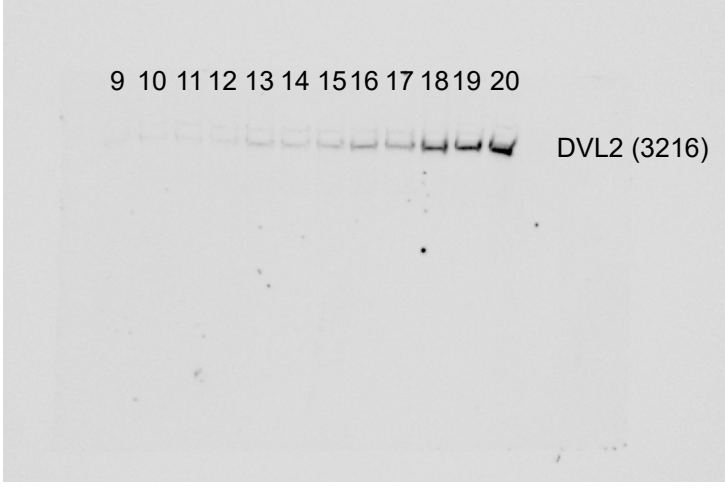

Supplement: Figure 1—figure supplement 1—source data 1. [file elife-96841-fig1-figsupp1-data1.zip › Figure 1-figure supplement 1-source data 1/Figure 1-Figure Supplement 1A and B.pdf]

To Panel C

unedited

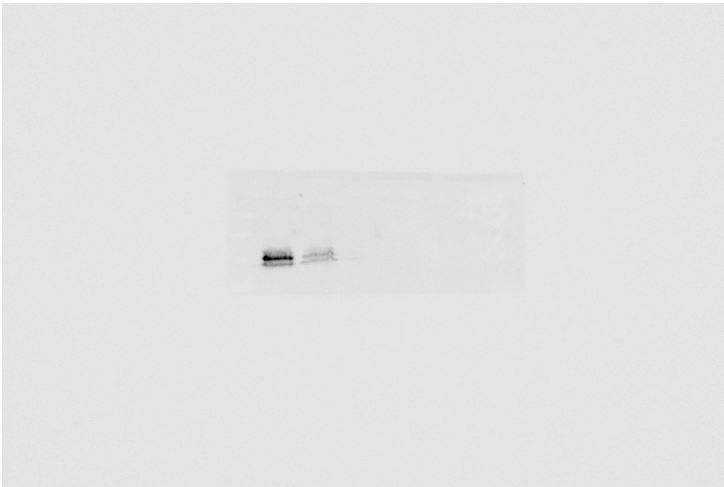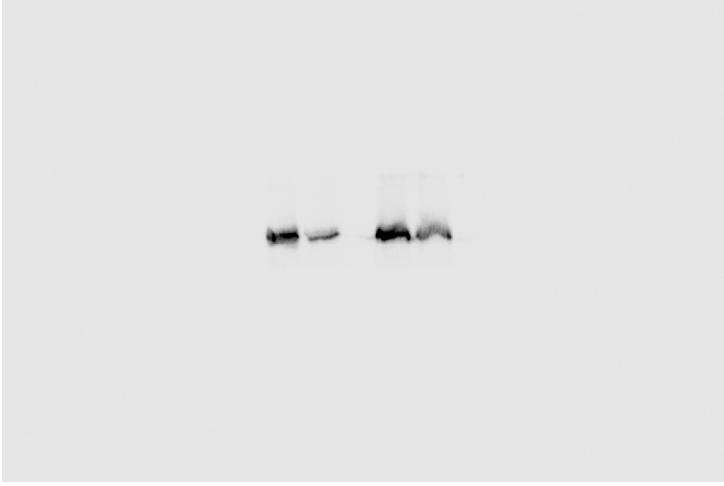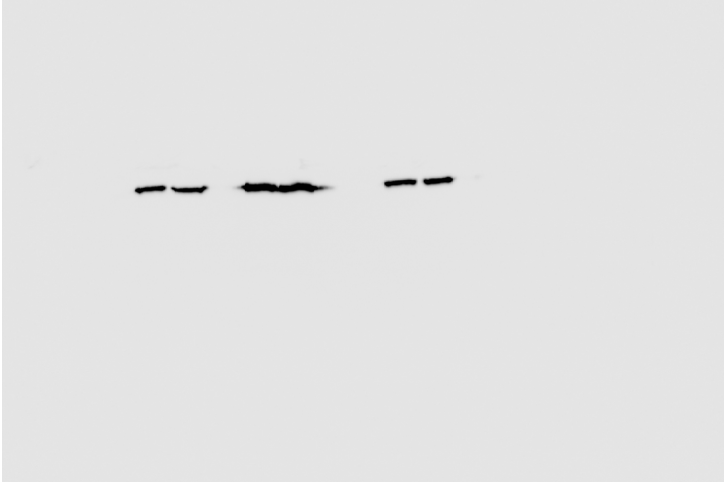

labelled

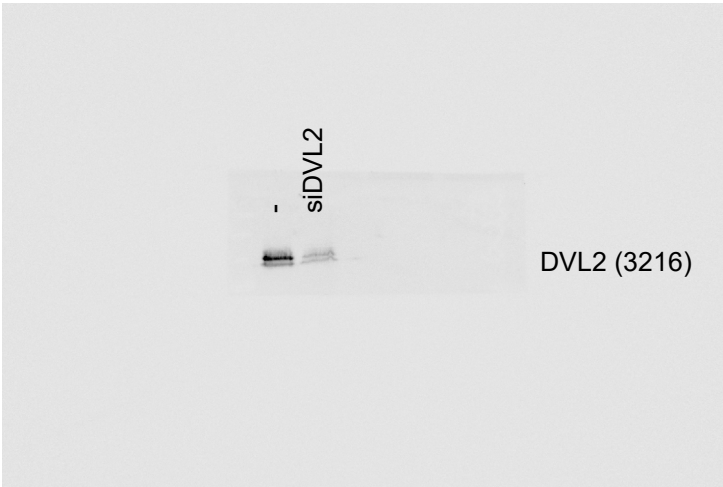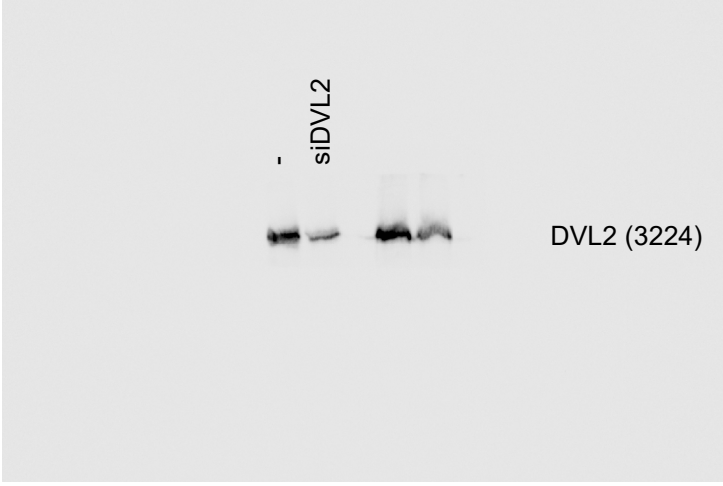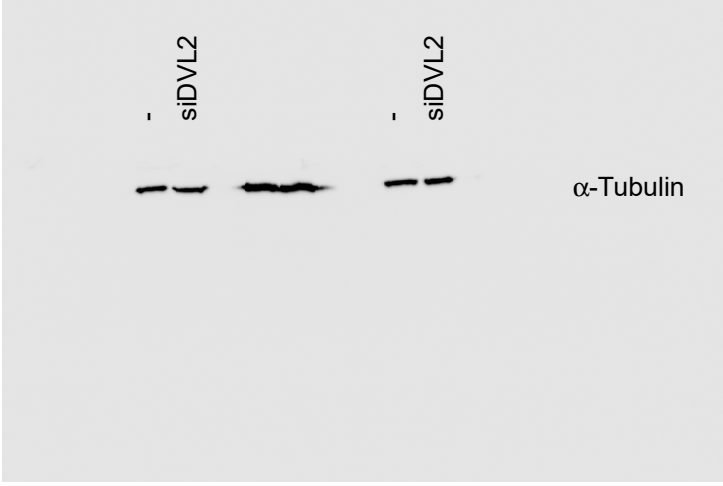

Supplement: Figure 1—figure supplement 1—source data 1. [file elife-96841-fig1-figsupp1-data1.zip › Figure 1-figure supplement 1-source data 1/Figure 1-Figure Supplement 1C.pdf]

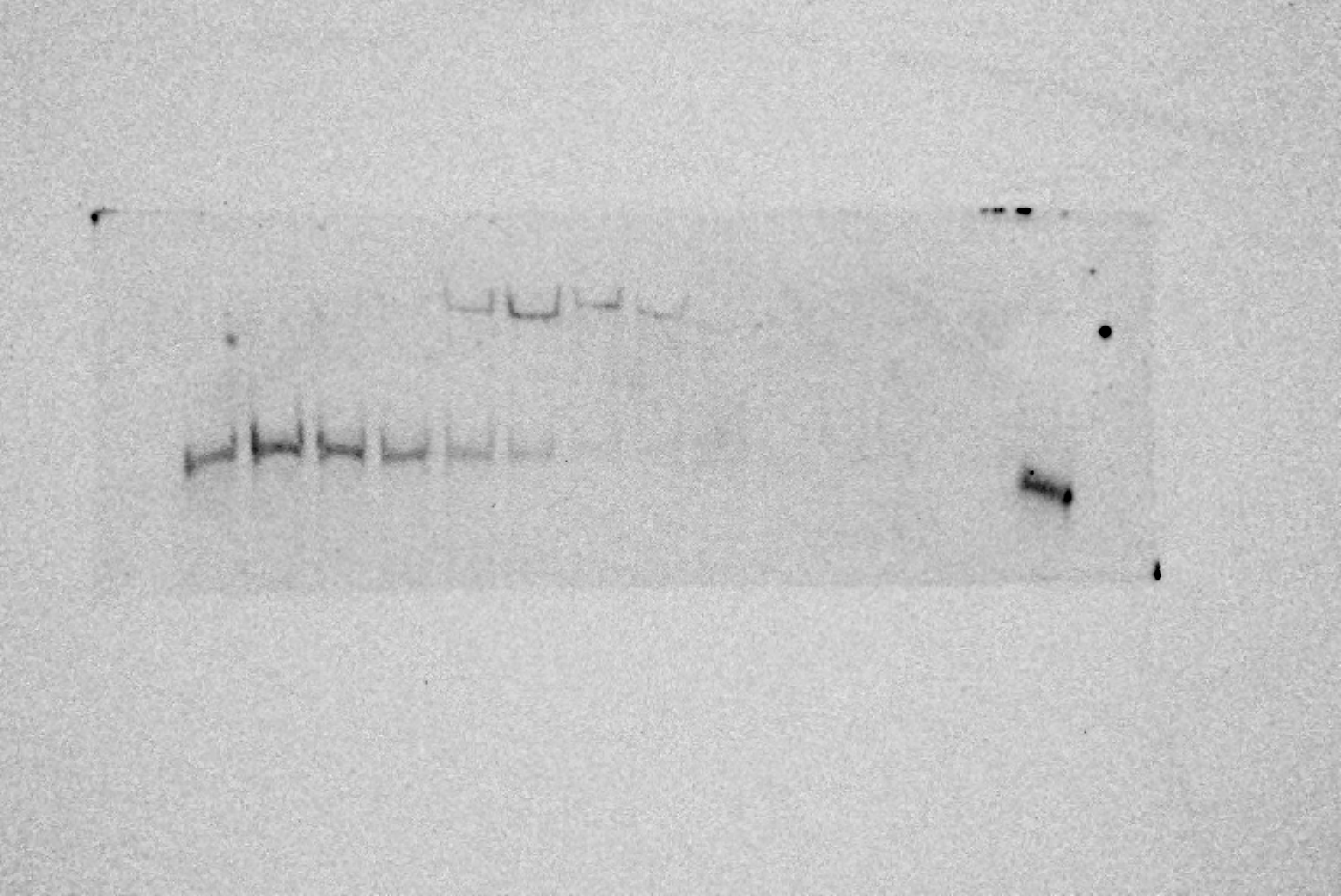

Supplement: Figure 1—figure supplement 1—source data 2. [file elife-96841-fig1-figsupp1-data2.zip › Figure 1-figure supplement 1-source data 2/Figure 1-figure supplement 1A - AXIN1.tif]

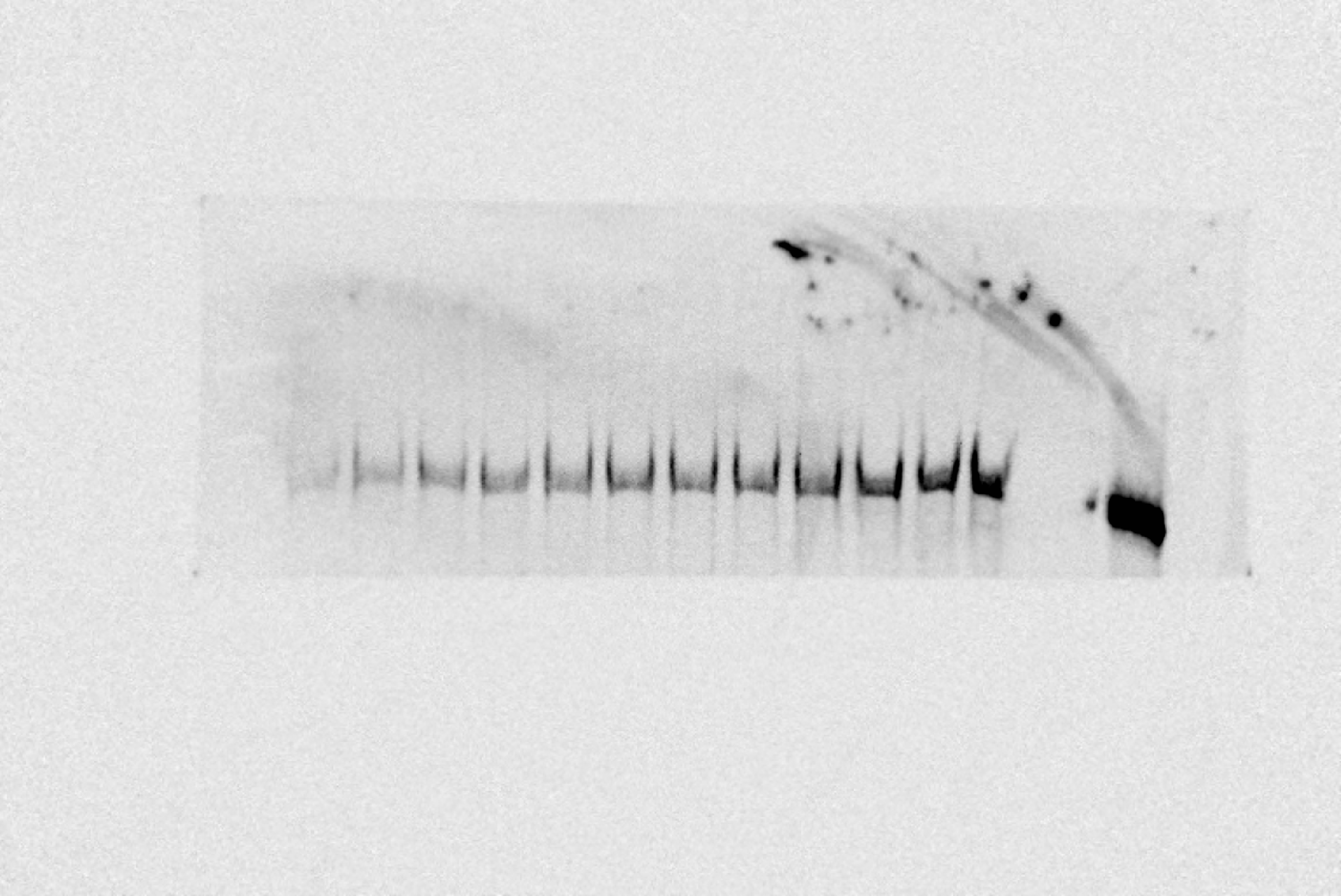

Supplement: Figure 1—figure supplement 1—source data 2. [file elife-96841-fig1-figsupp1-data2.zip › Figure 1-figure supplement 1-source data 2/Figure 1-figure supplement 1A - DVL2.tif]

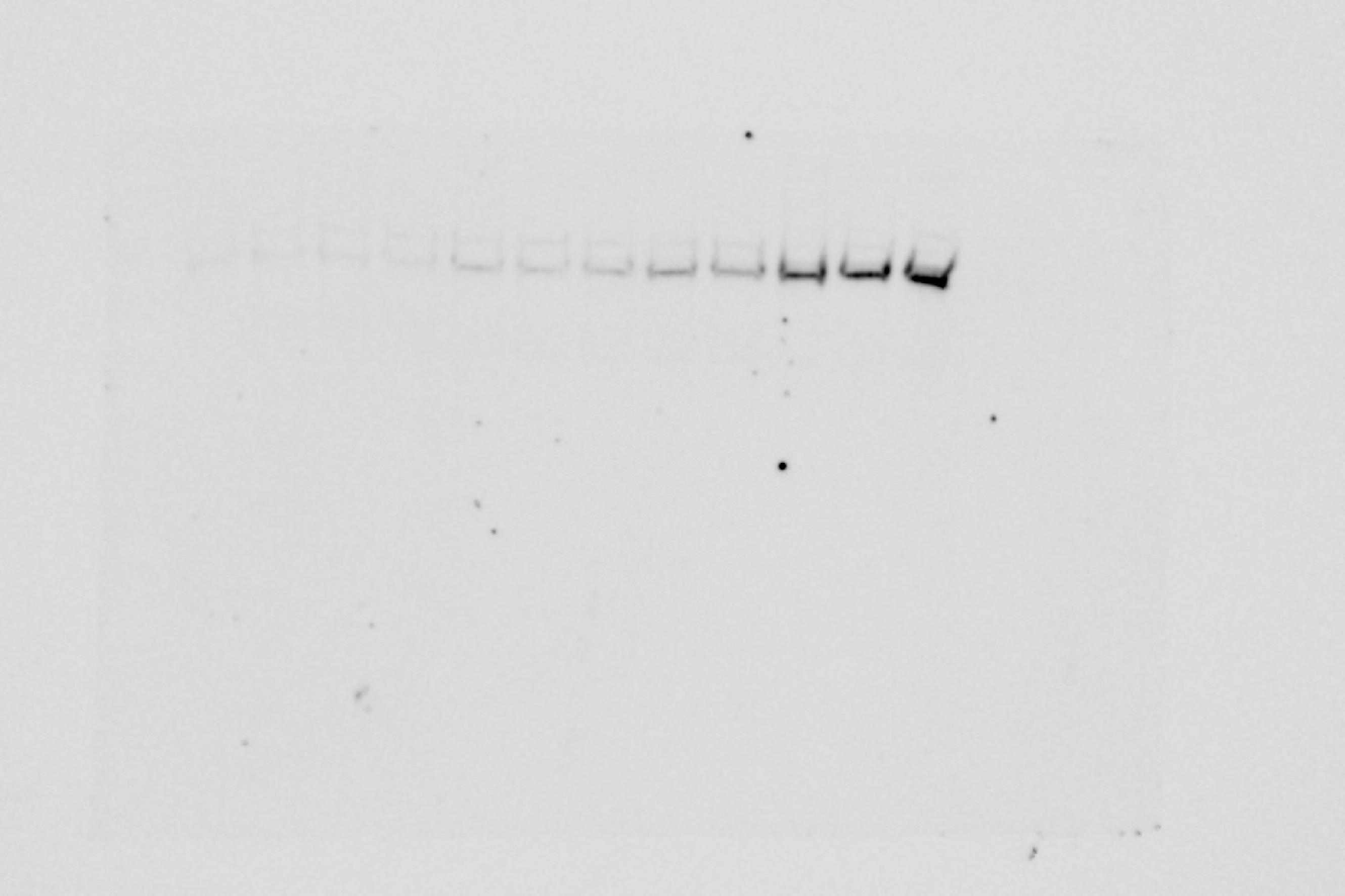

Supplement: Figure 1—figure supplement 1—source data 2. [file elife-96841-fig1-figsupp1-data2.zip › Figure 1-figure supplement 1-source data 2/Figure 1-figure supplement 1B - DVL2.tif]

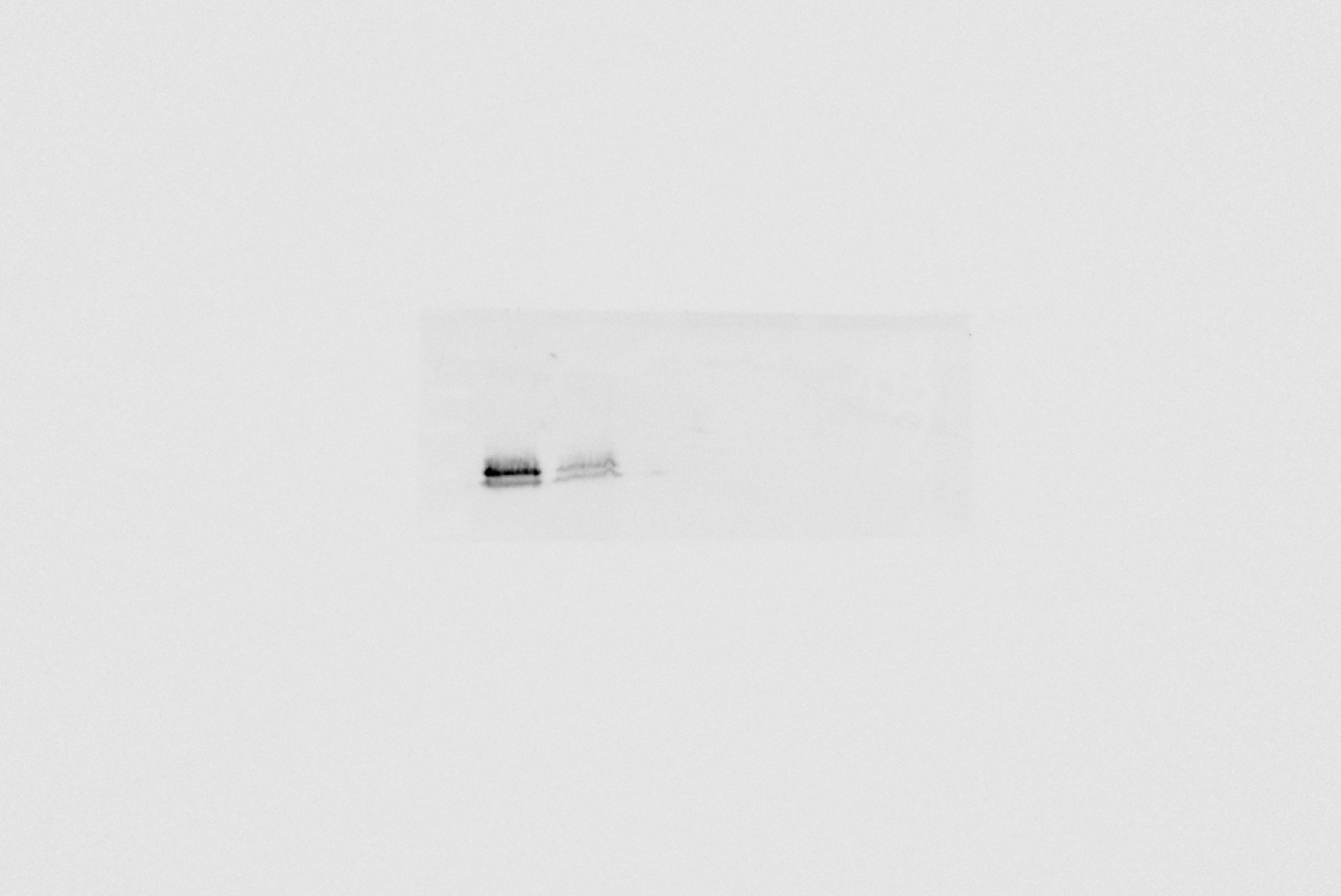

Supplement: Figure 1—figure supplement 1—source data 2. [file elife-96841-fig1-figsupp1-data2.zip › Figure 1-figure supplement 1-source data 2/Figure 1-figure supplement 1C - DVL2 3216.tif]

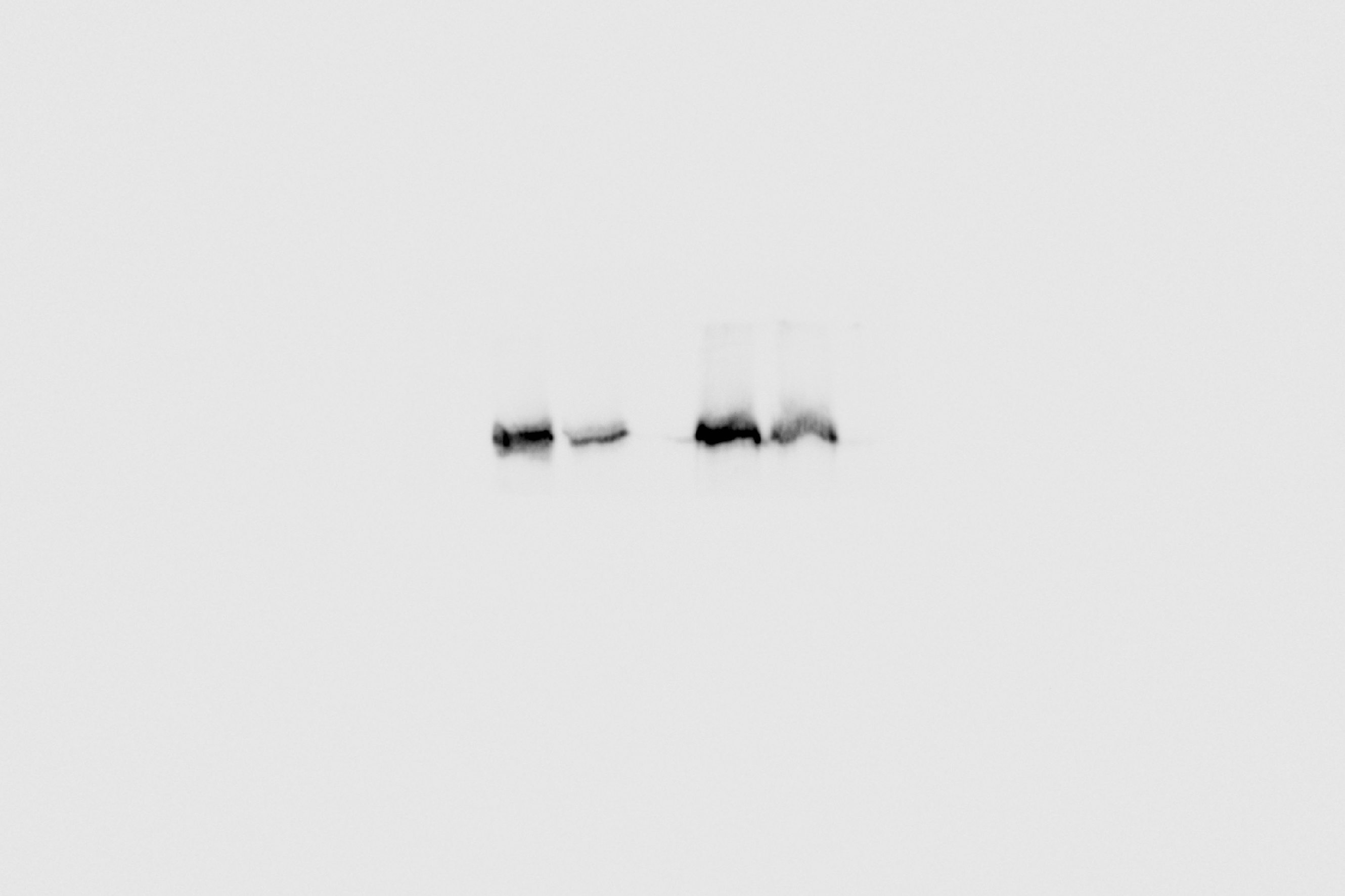

Supplement: Figure 1—figure supplement 1—source data 2. [file elife-96841-fig1-figsupp1-data2.zip › Figure 1-figure supplement 1-source data 2/Figure 1-figure supplement 1C - DVL2 3224.tif]

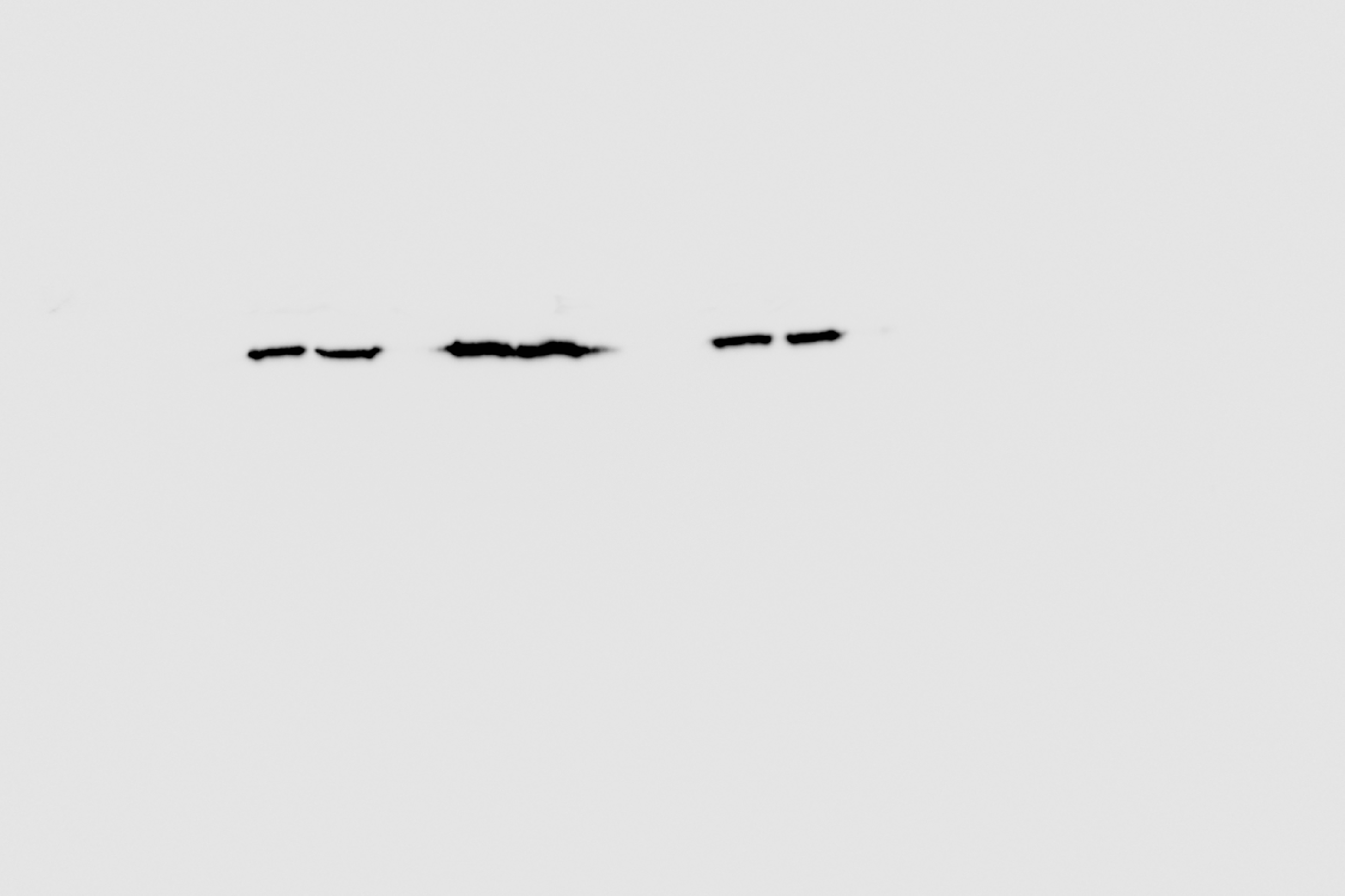

Supplement: Figure 1—figure supplement 1—source data 2. [file elife-96841-fig1-figsupp1-data2.zip › Figure 1-figure supplement 1-source data 2/Figure 1-figure supplement 1C - Tubulin.tif]

To Panel B

unedited

labelled

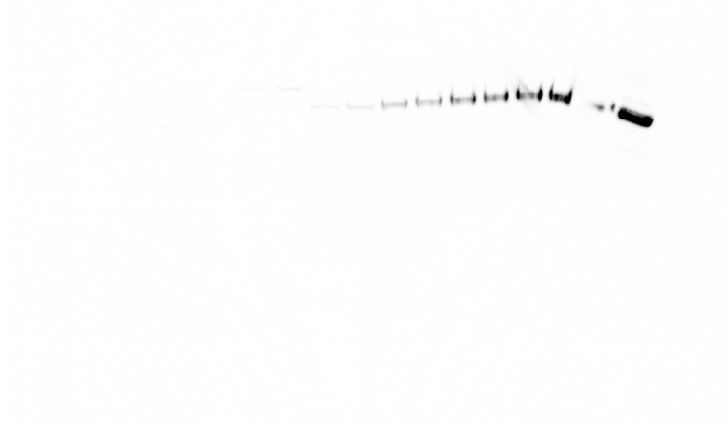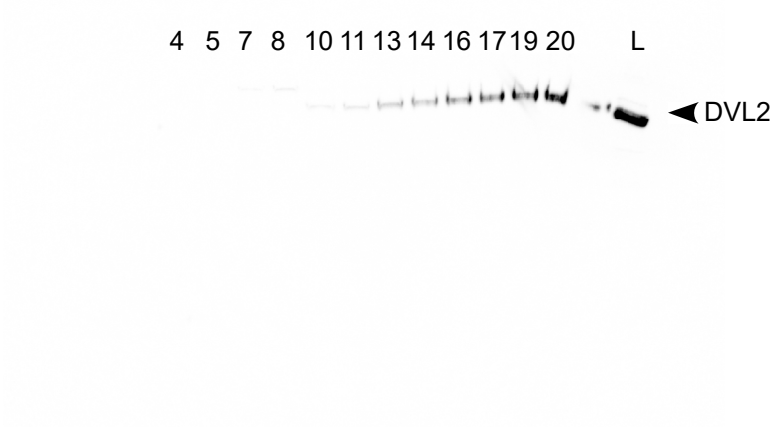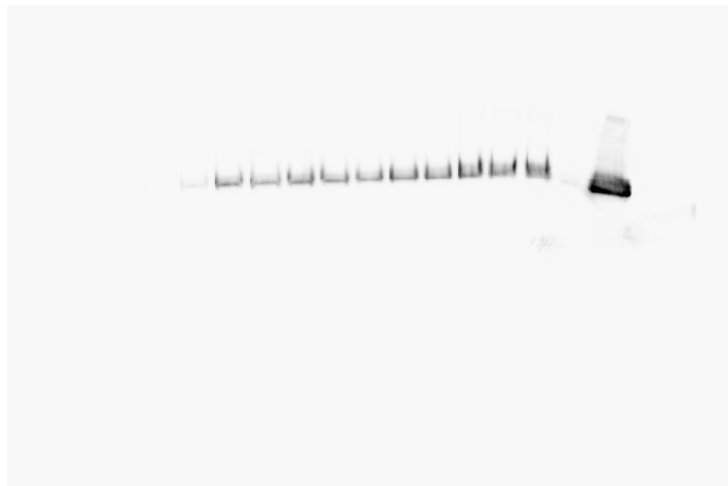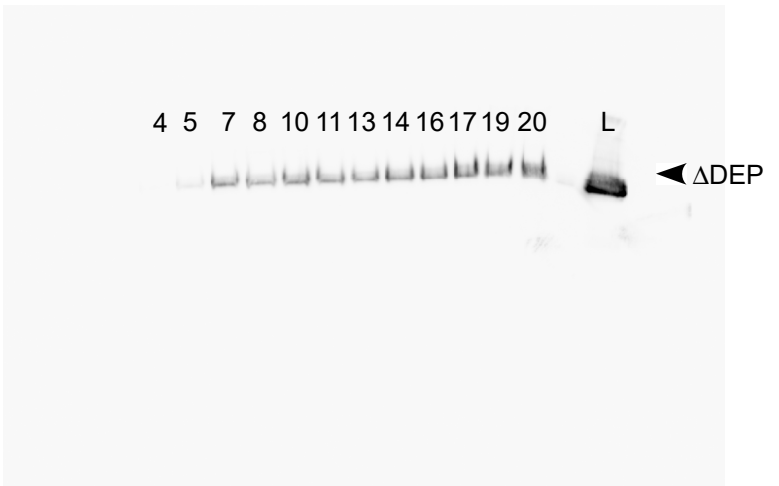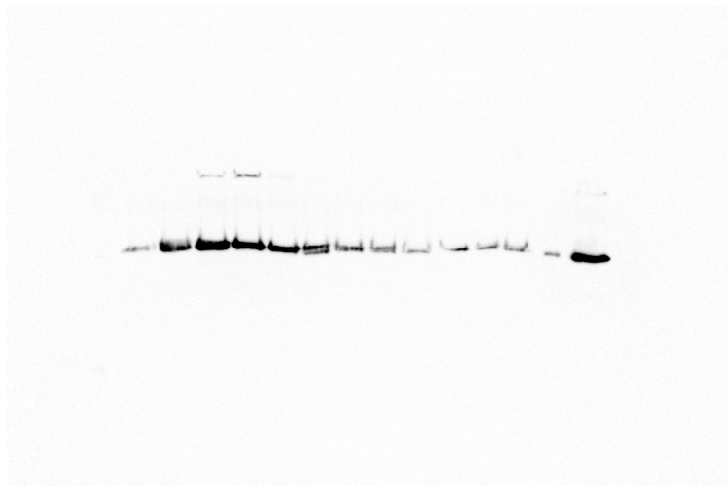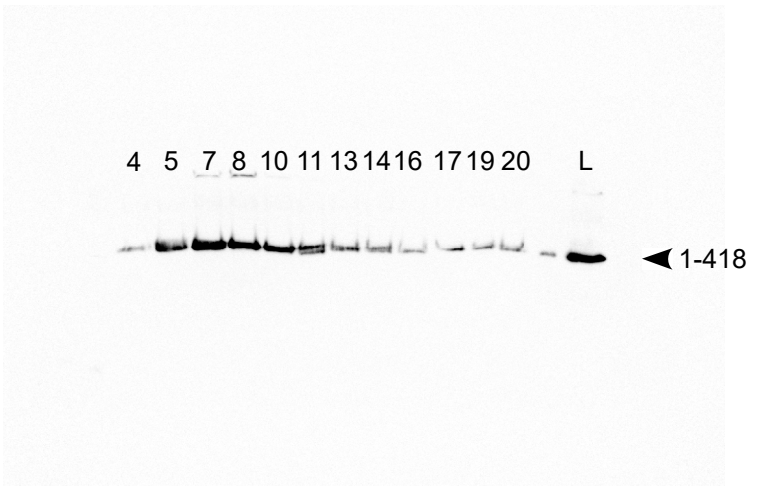

Supplement: Figure 2—source data 2. [file elife-96841-fig2-data2.zip › Figure 2-source data 2/Figure 2B.pdf]

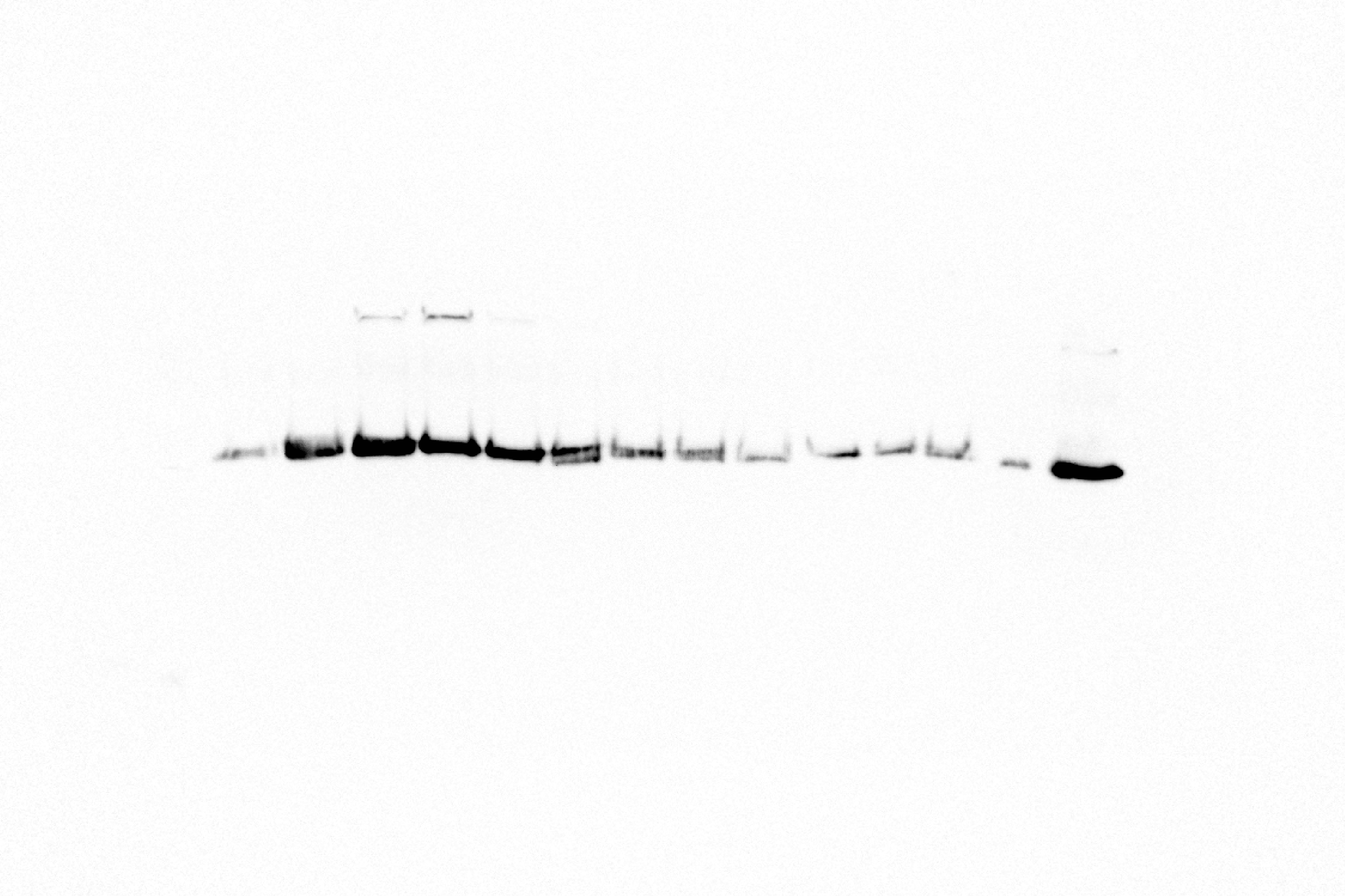

Supplement: Figure 2—source data 3. [file elife-96841-fig2-data3.zip › Figure 2-source data 3/Figure 2B - 1-418.tif]

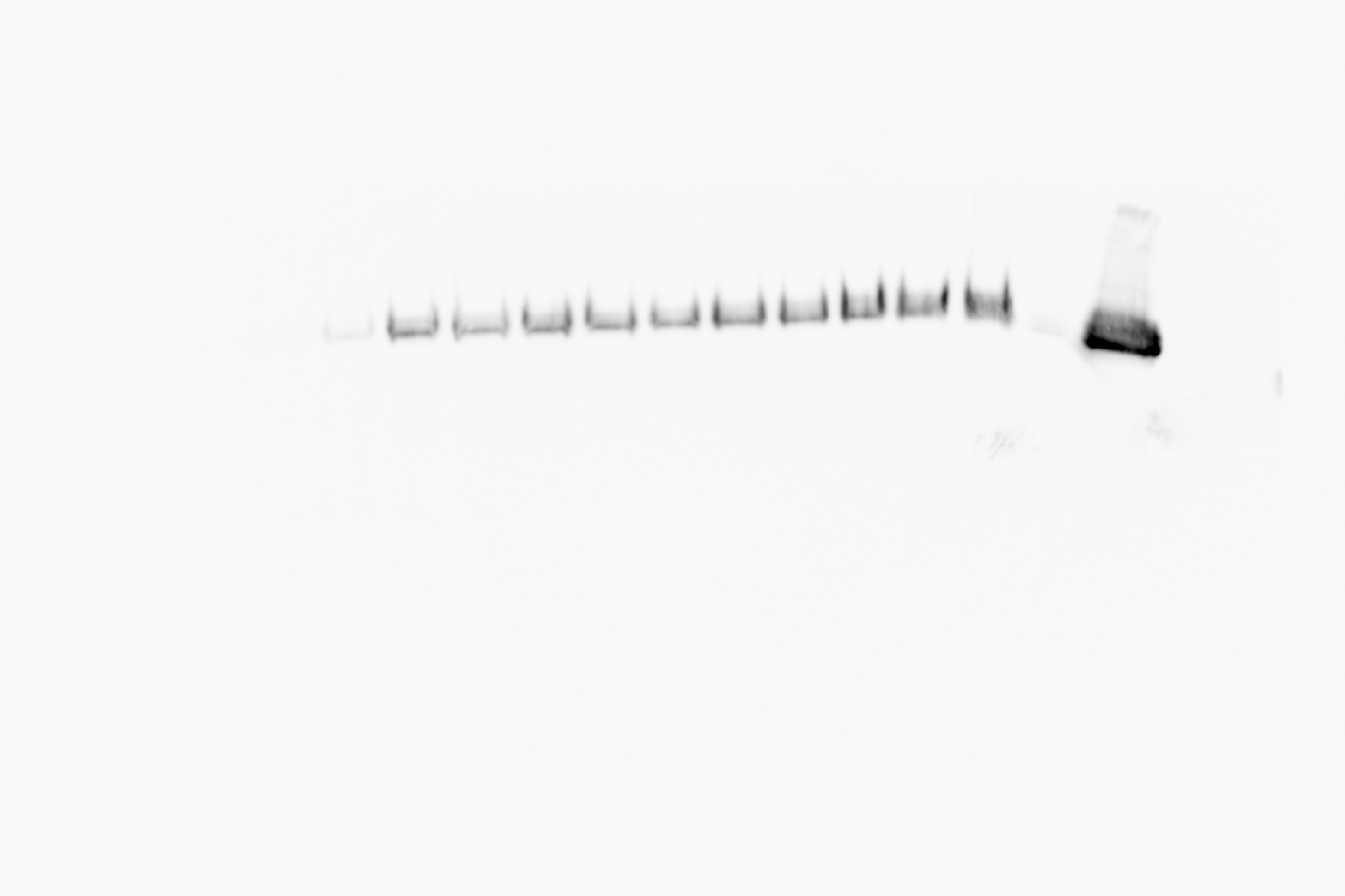

Supplement: Figure 2—source data 3. [file elife-96841-fig2-data3.zip › Figure 2-source data 3/Figure 2B - deltaDEP.tif]

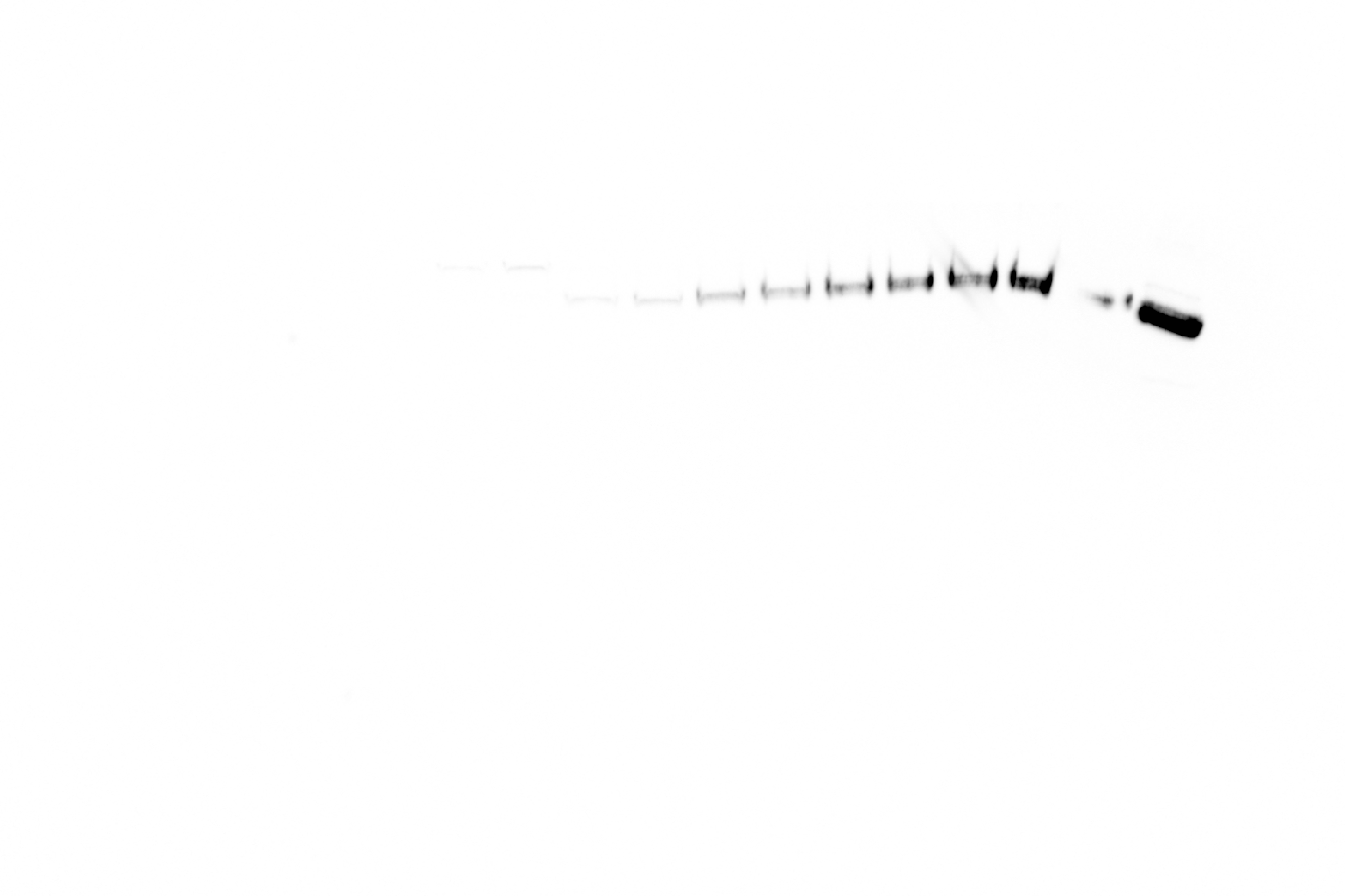

Supplement: Figure 2—source data 3. [file elife-96841-fig2-data3.zip › Figure 2-source data 3/Figure 2B - DVL2.tif]

To Panel A

unedited

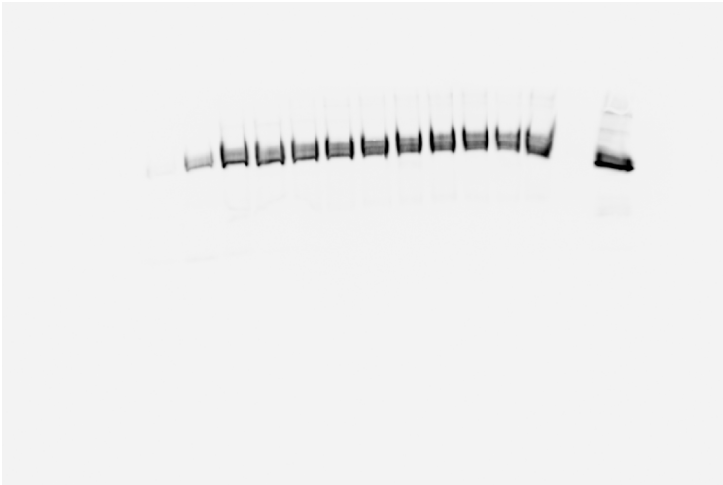

labelled

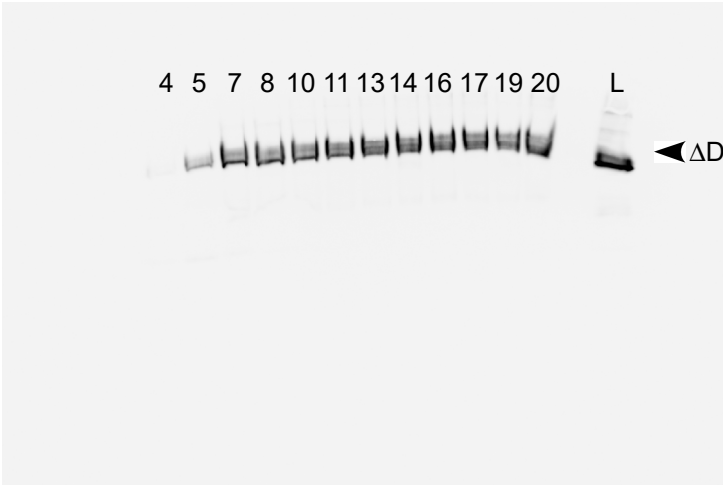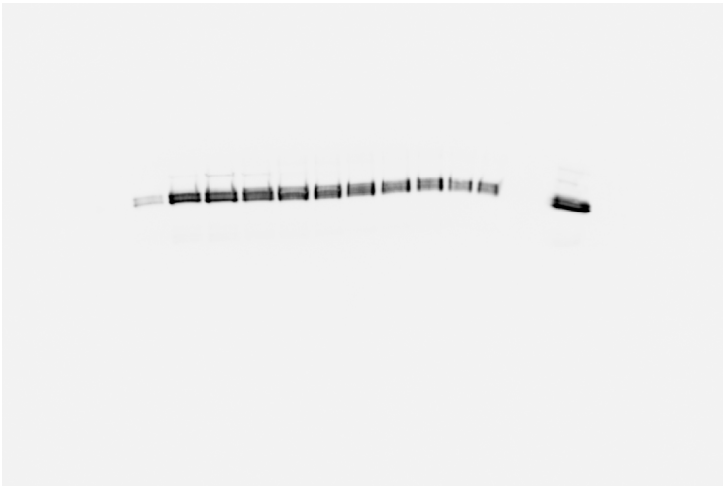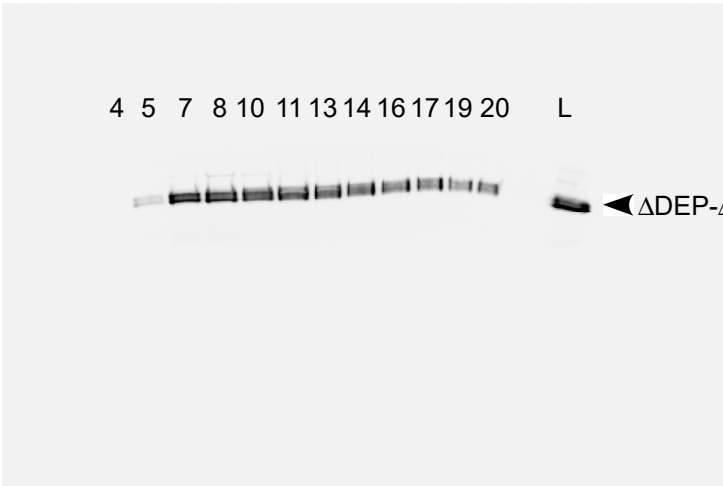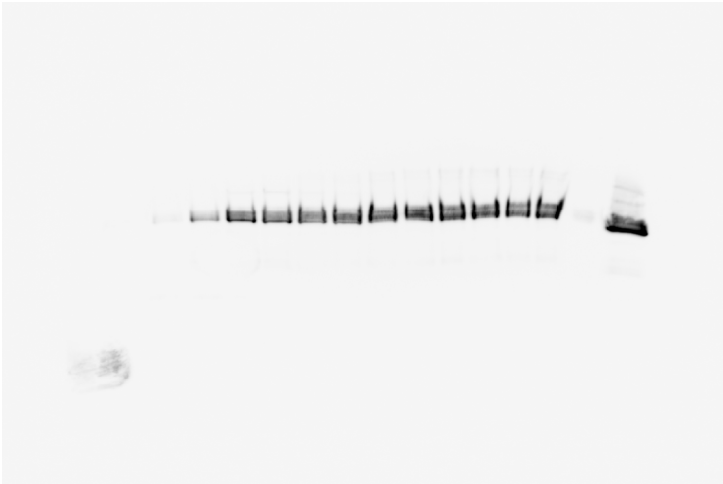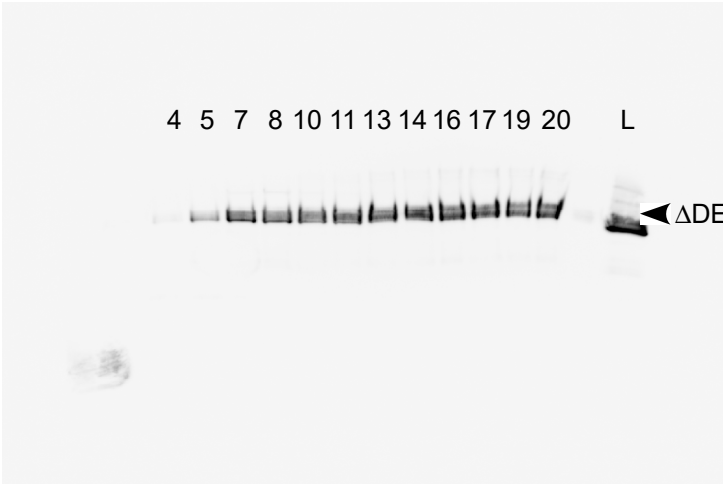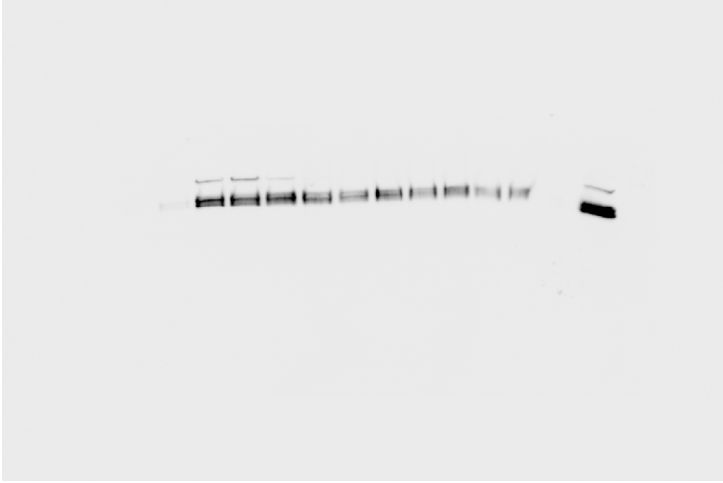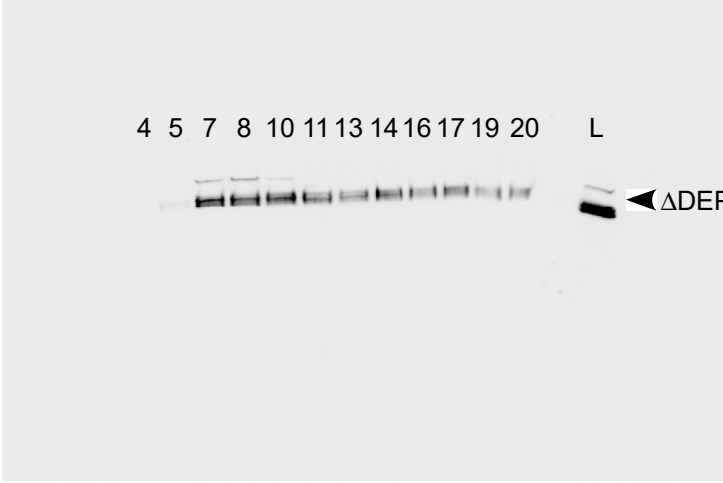

Supplement: Figure 4—source data 2. [file elife-96841-fig4-data2.zip › Figure 4-source data 2/Figure 4A.pdf]

To Panel C

unedited

labelled

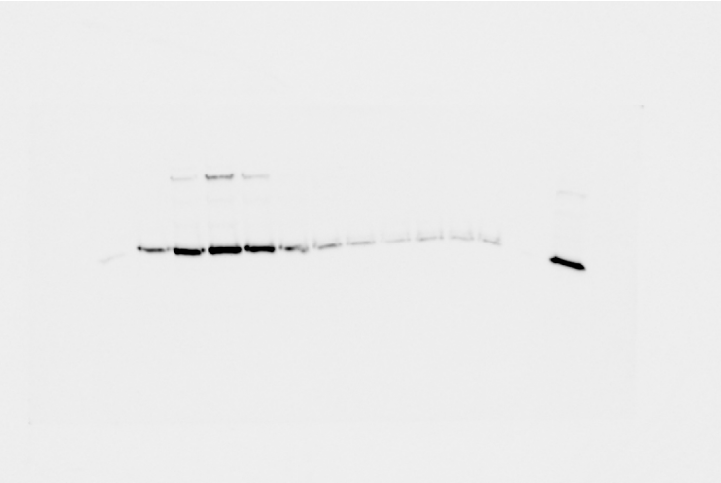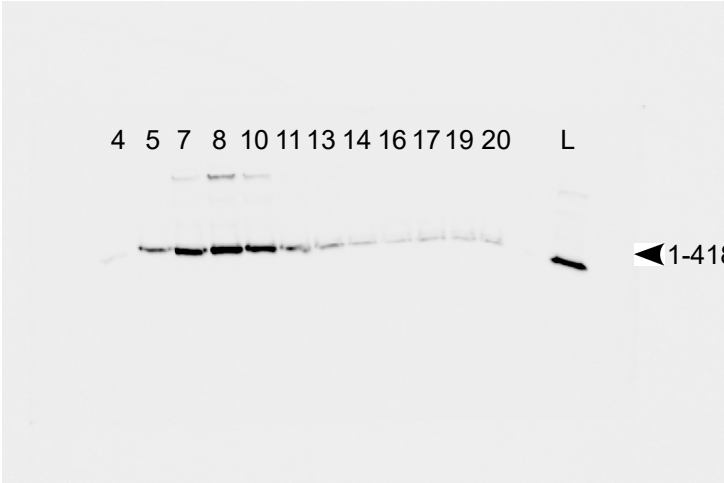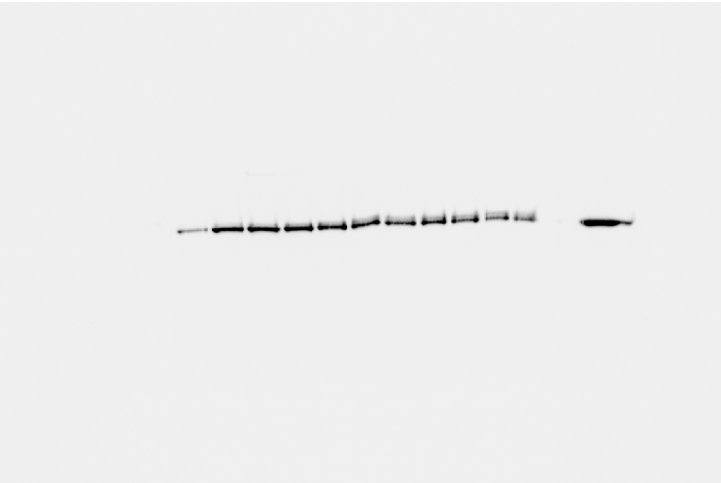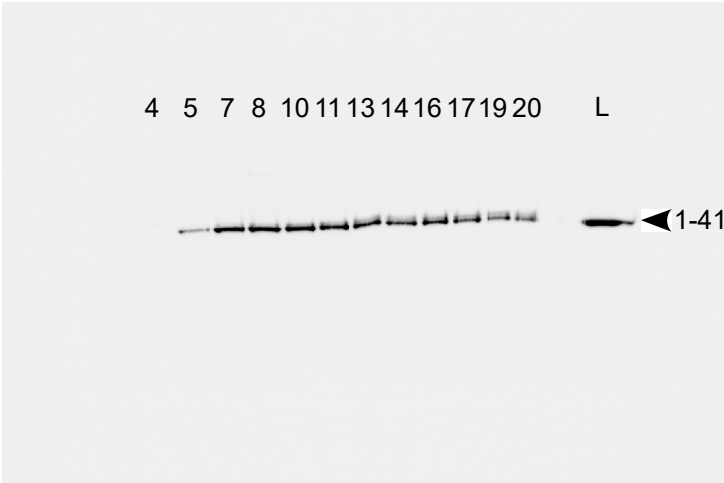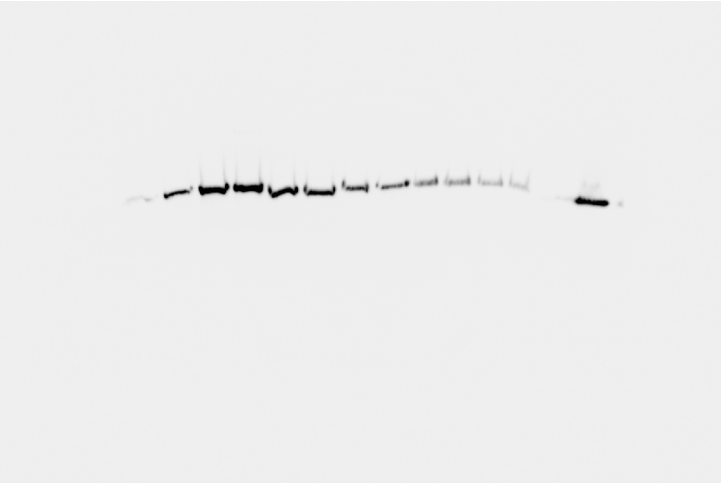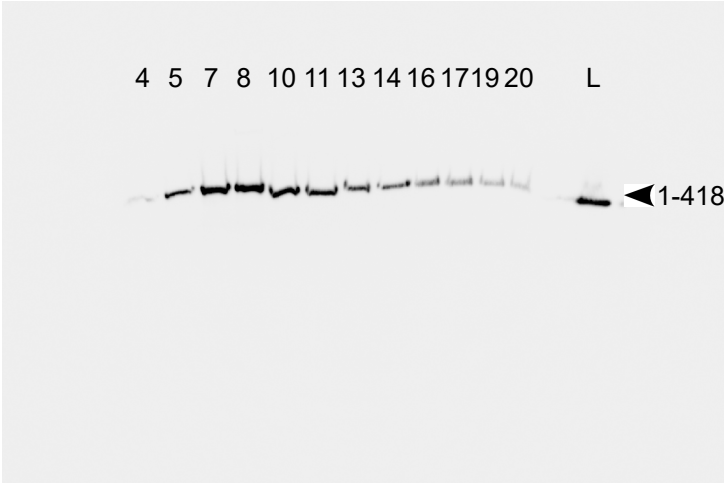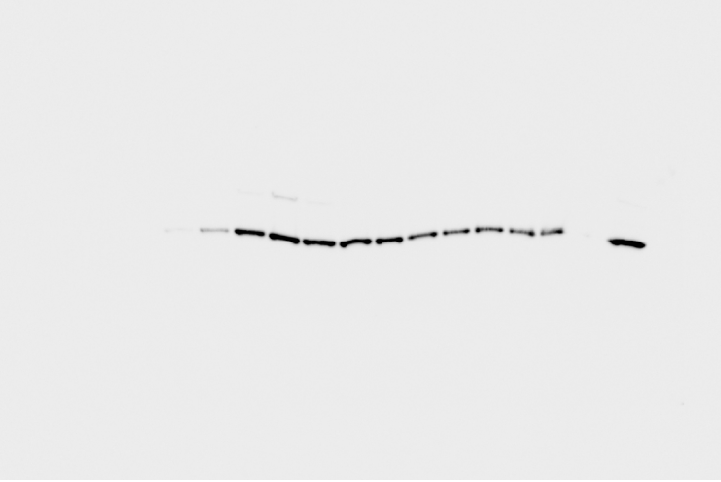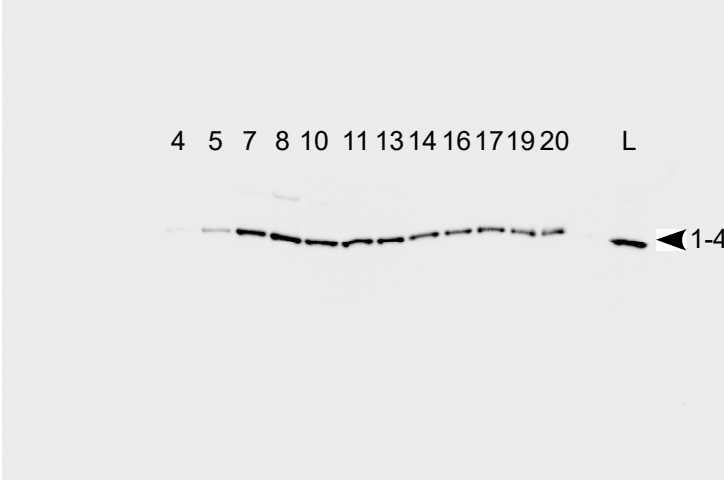

Supplement: Figure 4—source data 2. [file elife-96841-fig4-data2.zip › Figure 4-source data 2/Figure 4C.pdf]

To Panel E

unedited

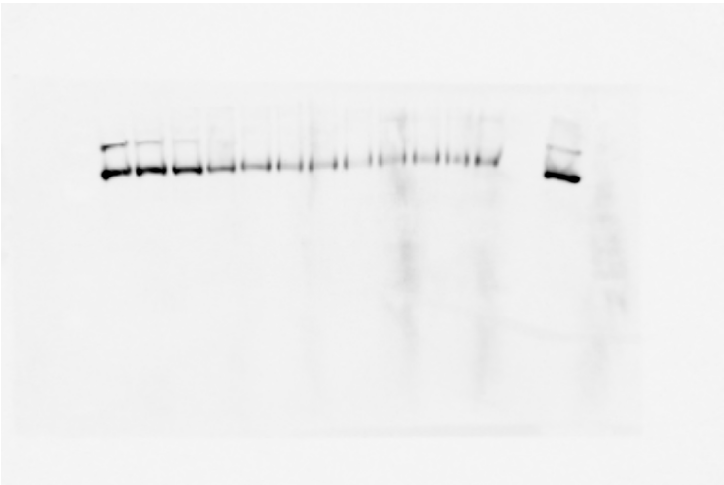

labelled

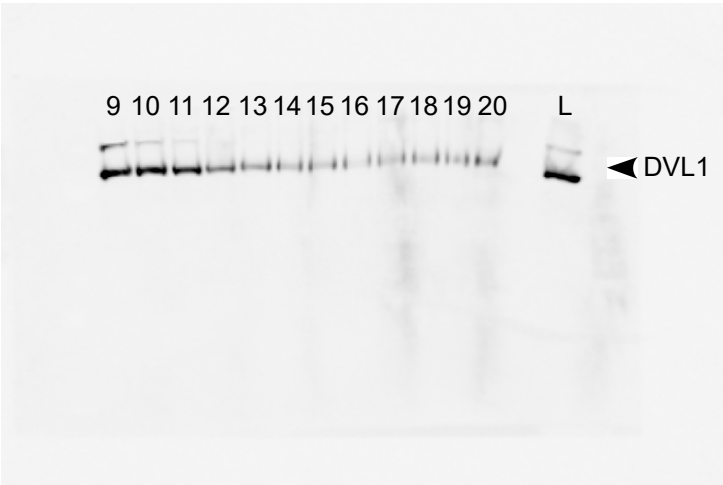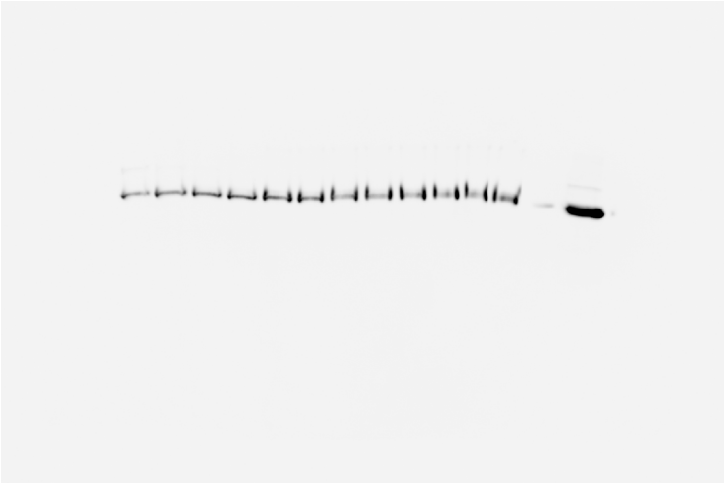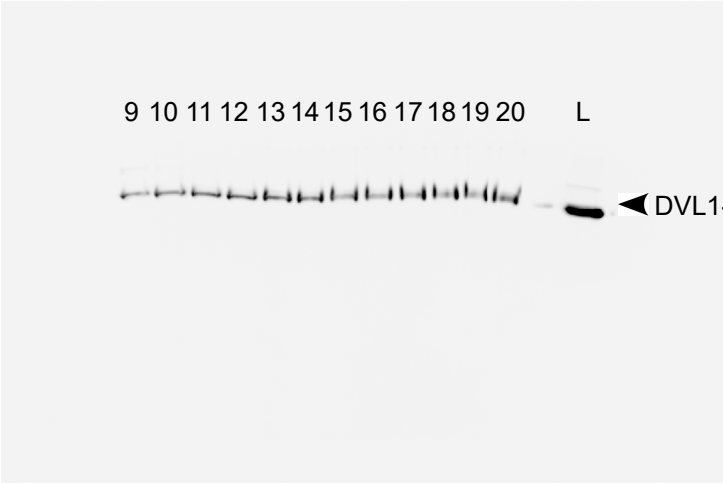

Supplement: Figure 4—source data 2. [file elife-96841-fig4-data2.zip › Figure 4-source data 2/Figure 4E.pdf]

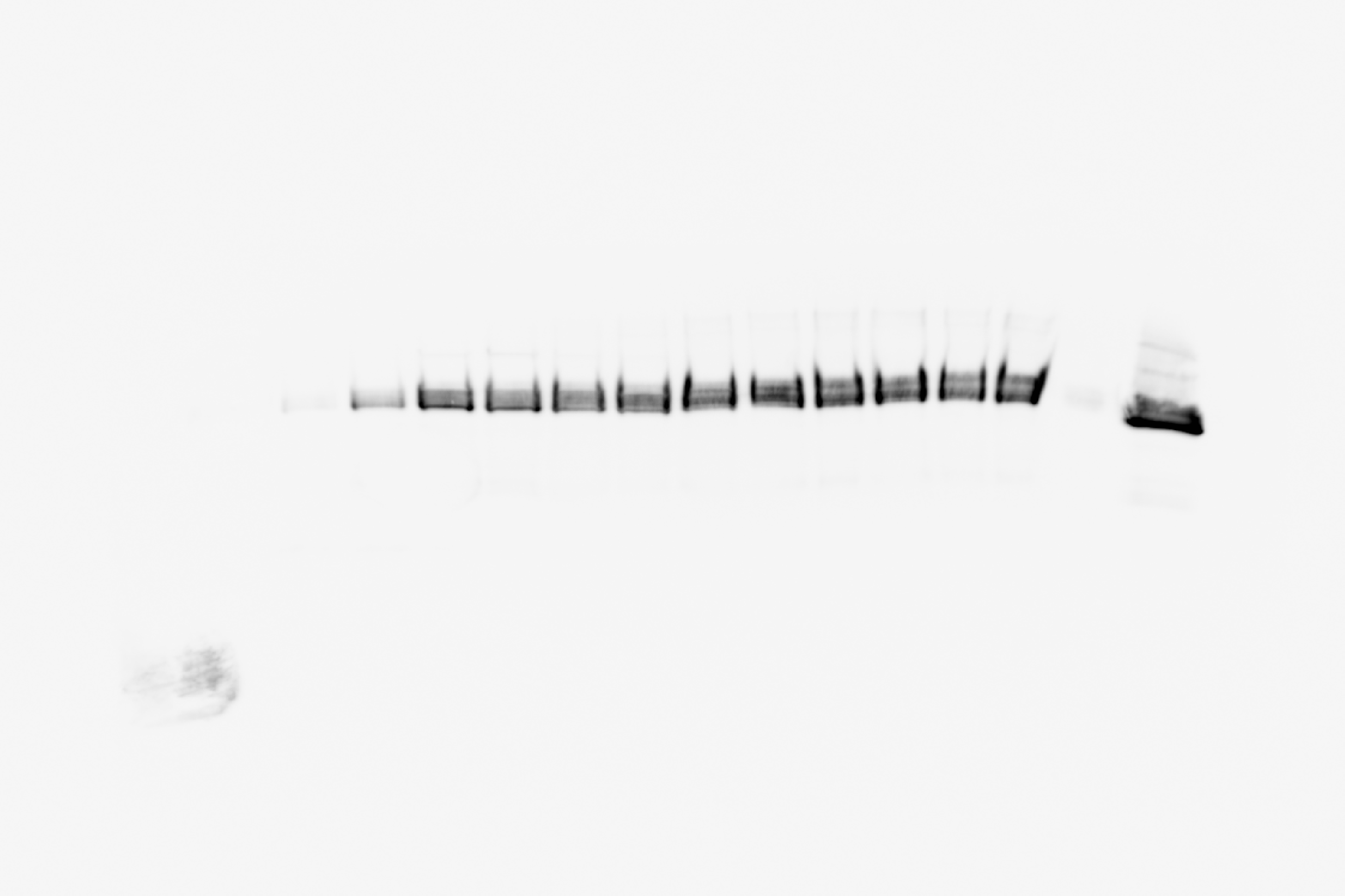

Supplement: Figure 4—source data 3. [file elife-96841-fig4-data3.zip › Figure 4-source data 3/Figure 4A - deltaDEP-deltaCD2.tif]

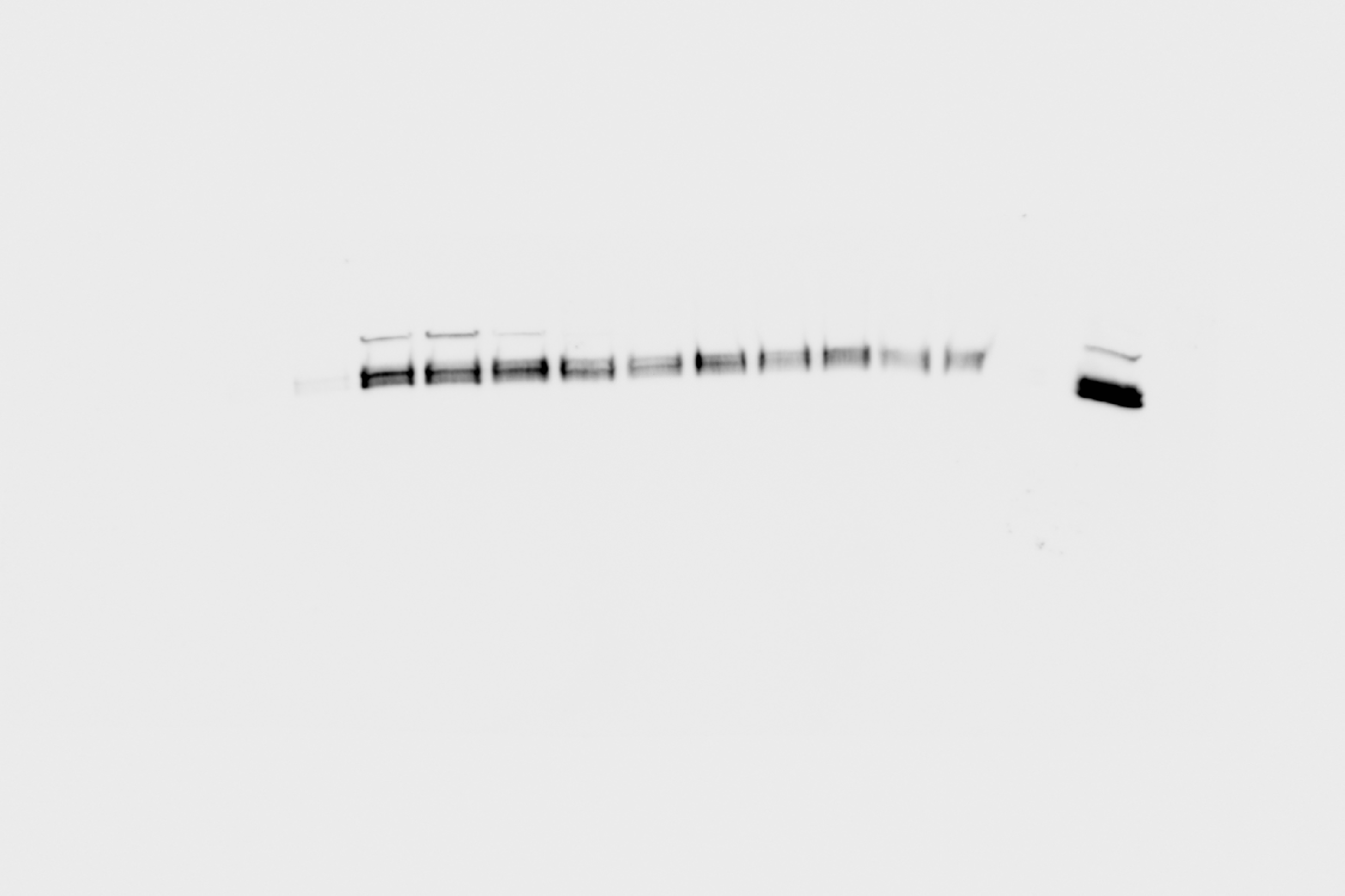

Supplement: Figure 4—source data 3. [file elife-96841-fig4-data3.zip › Figure 4-source data 3/Figure 4A - deltaDEP-deltaCFR.tif]

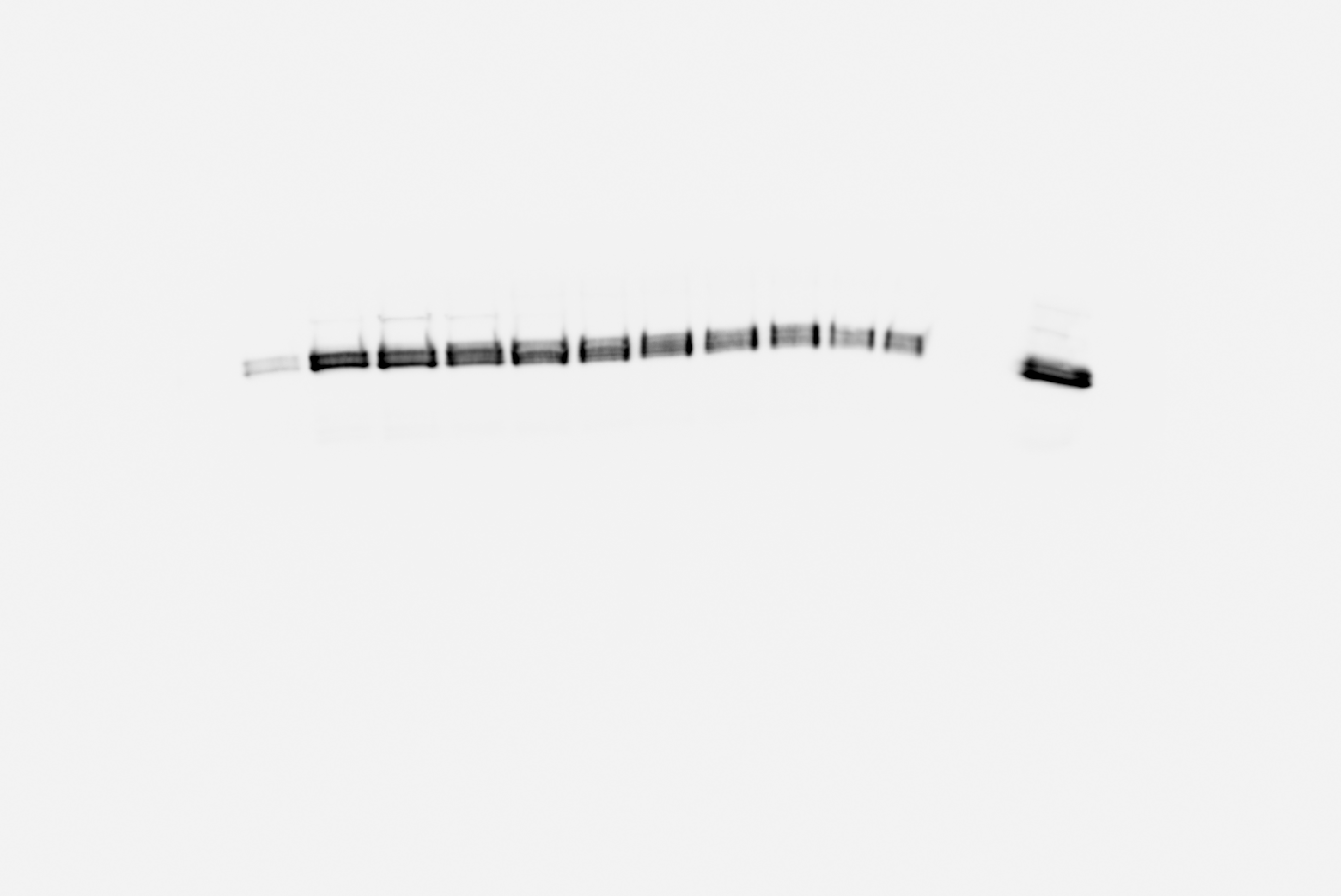

Supplement: Figure 4—source data 3. [file elife-96841-fig4-data3.zip › Figure 4-source data 3/Figure 4A - deltaDEP-deltaLCR4.tif]

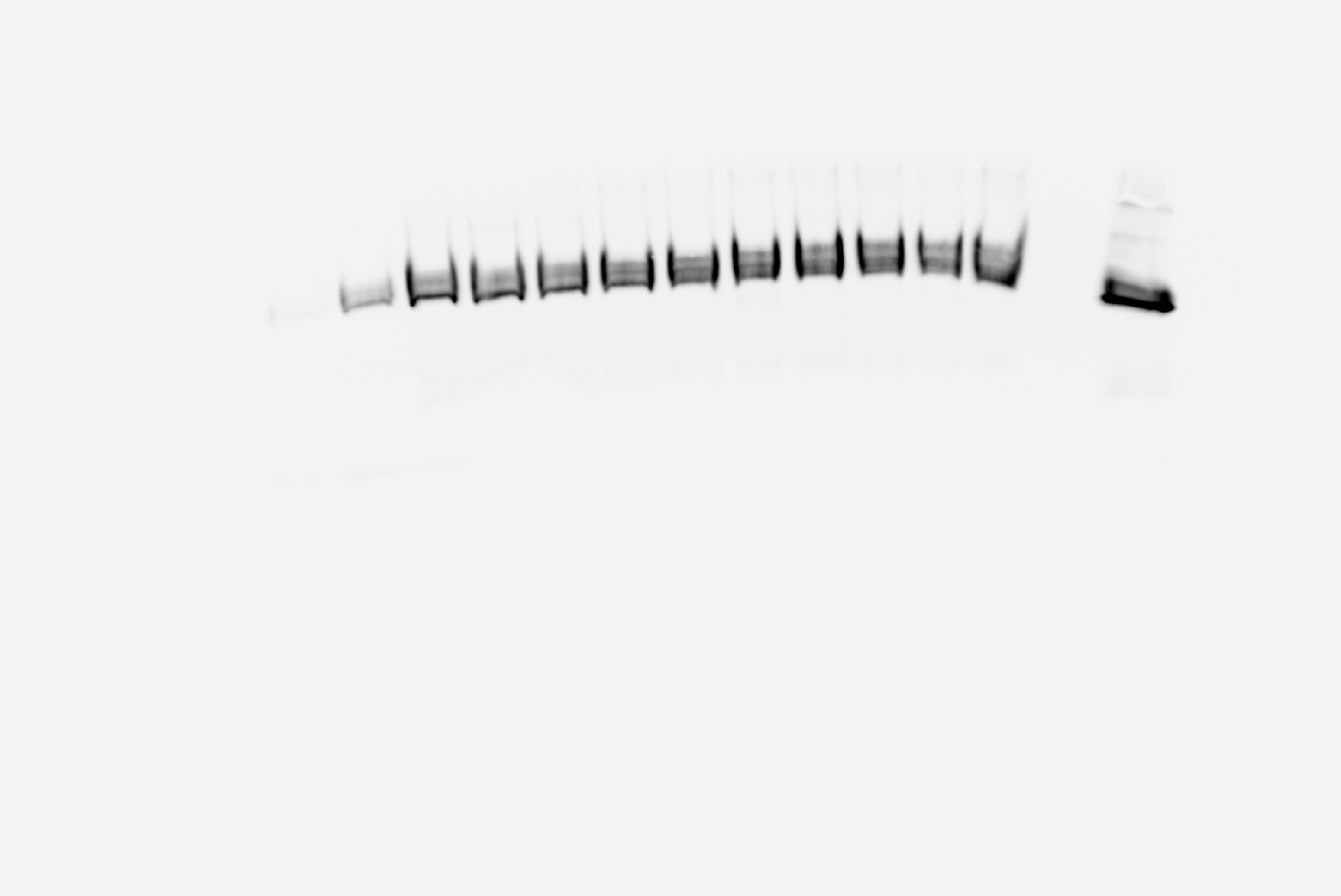

Supplement: Figure 4—source data 3. [file elife-96841-fig4-data3.zip › Figure 4-source data 3/Figure 4A - deltaDEP.tif]

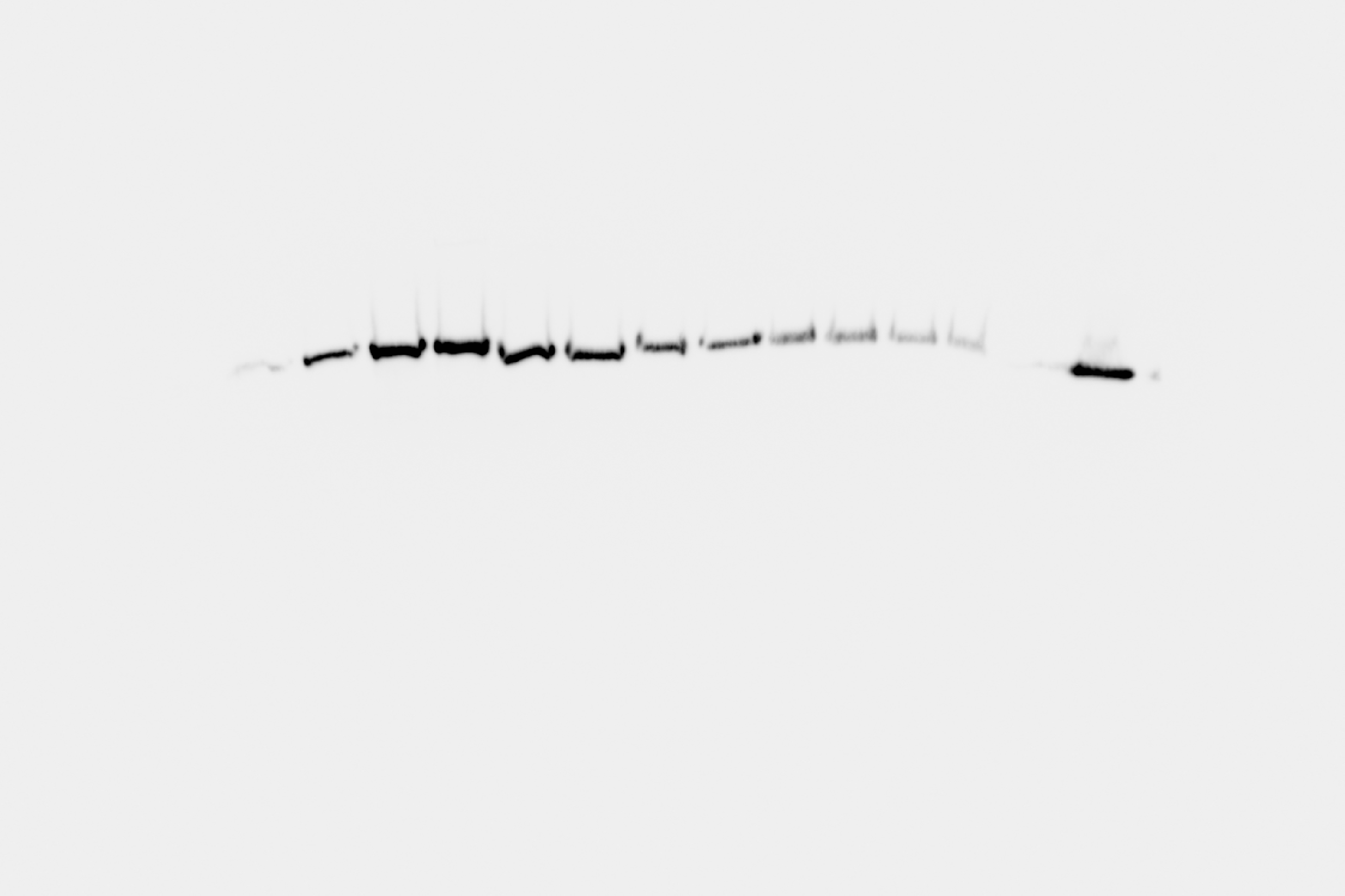

Supplement: Figure 4—source data 3. [file elife-96841-fig4-data3.zip › Figure 4-source data 3/Figure 4C - 1-418+CD2.tif]

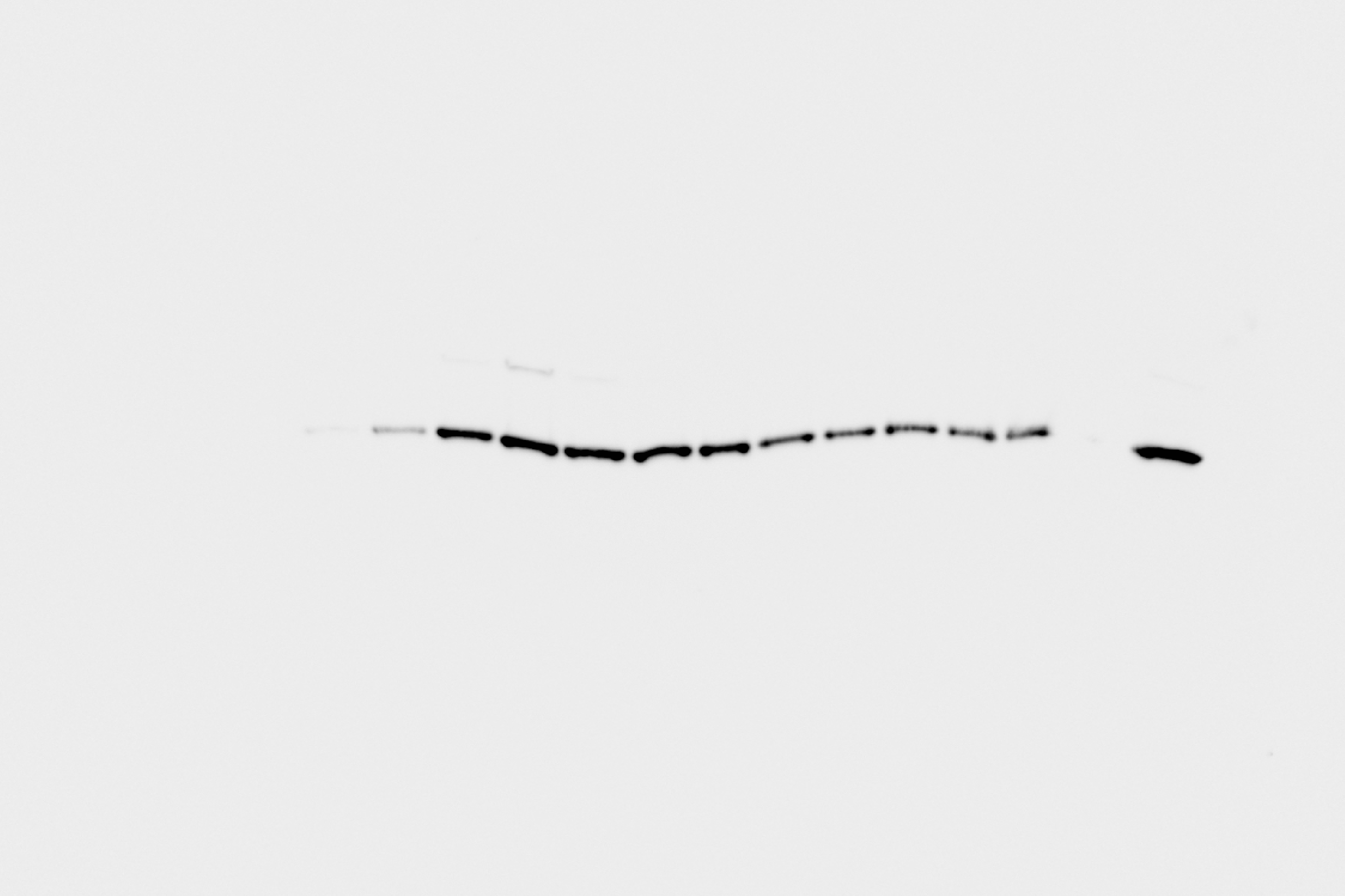

Supplement: Figure 4—source data 3. [file elife-96841-fig4-data3.zip › Figure 4-source data 3/Figure 4C - 1-418+CFR.tif]

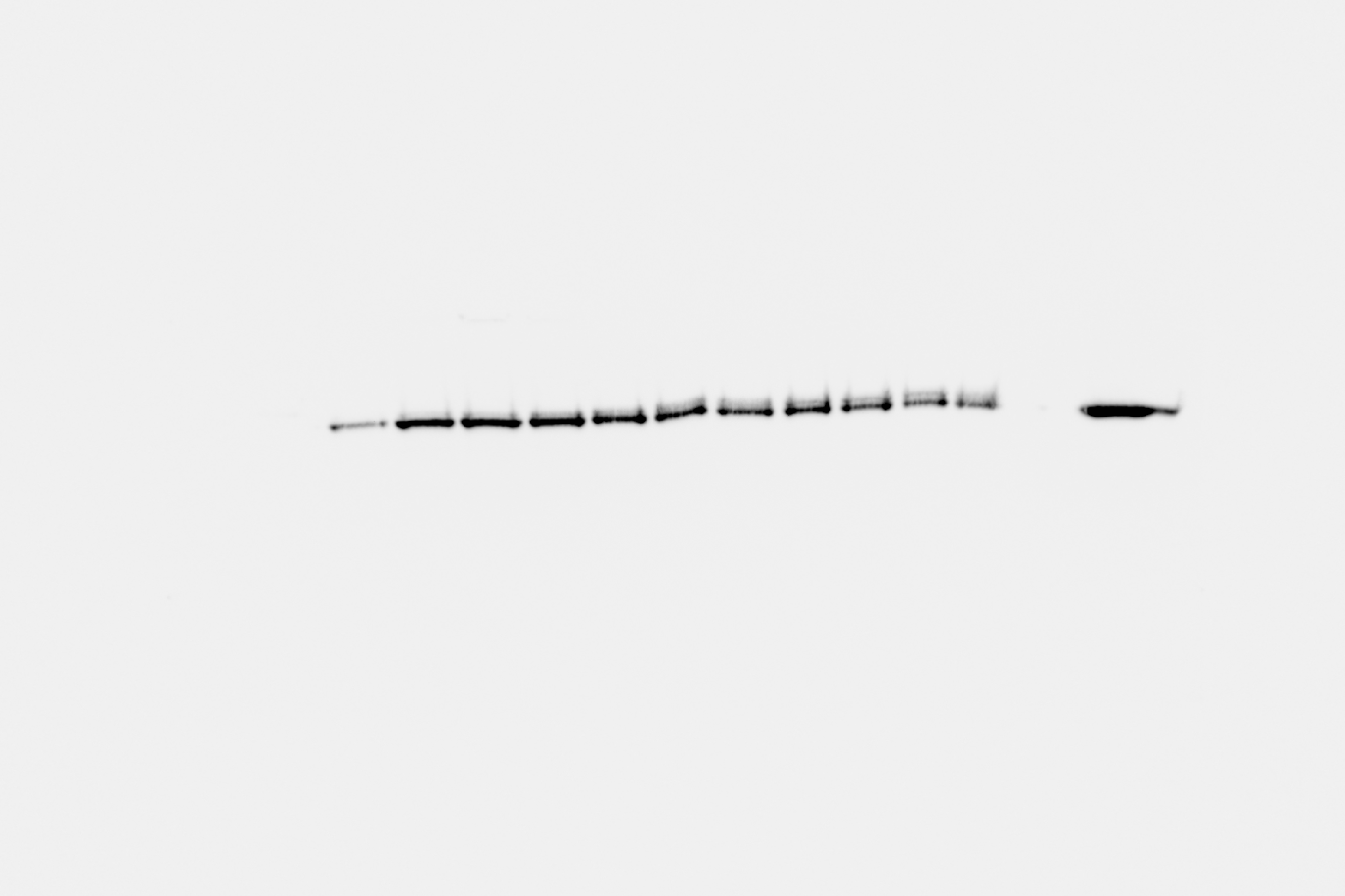

Supplement: Figure 4—source data 3. [file elife-96841-fig4-data3.zip › Figure 4-source data 3/Figure 4C - 1-418+LCR4.tif]

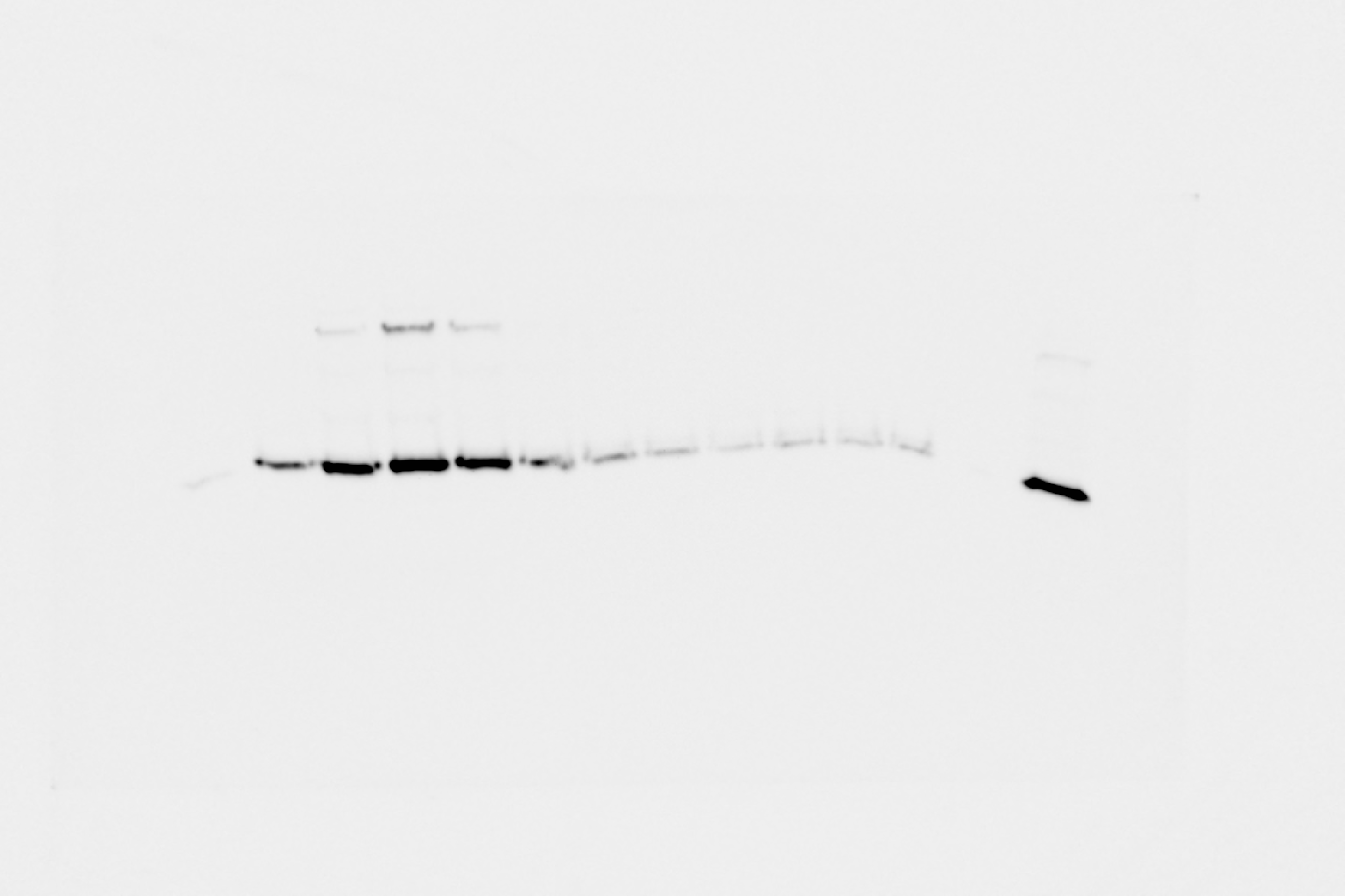

Supplement: Figure 4—source data 3. [file elife-96841-fig4-data3.zip › Figure 4-source data 3/Figure 4C - 1-418.tif]

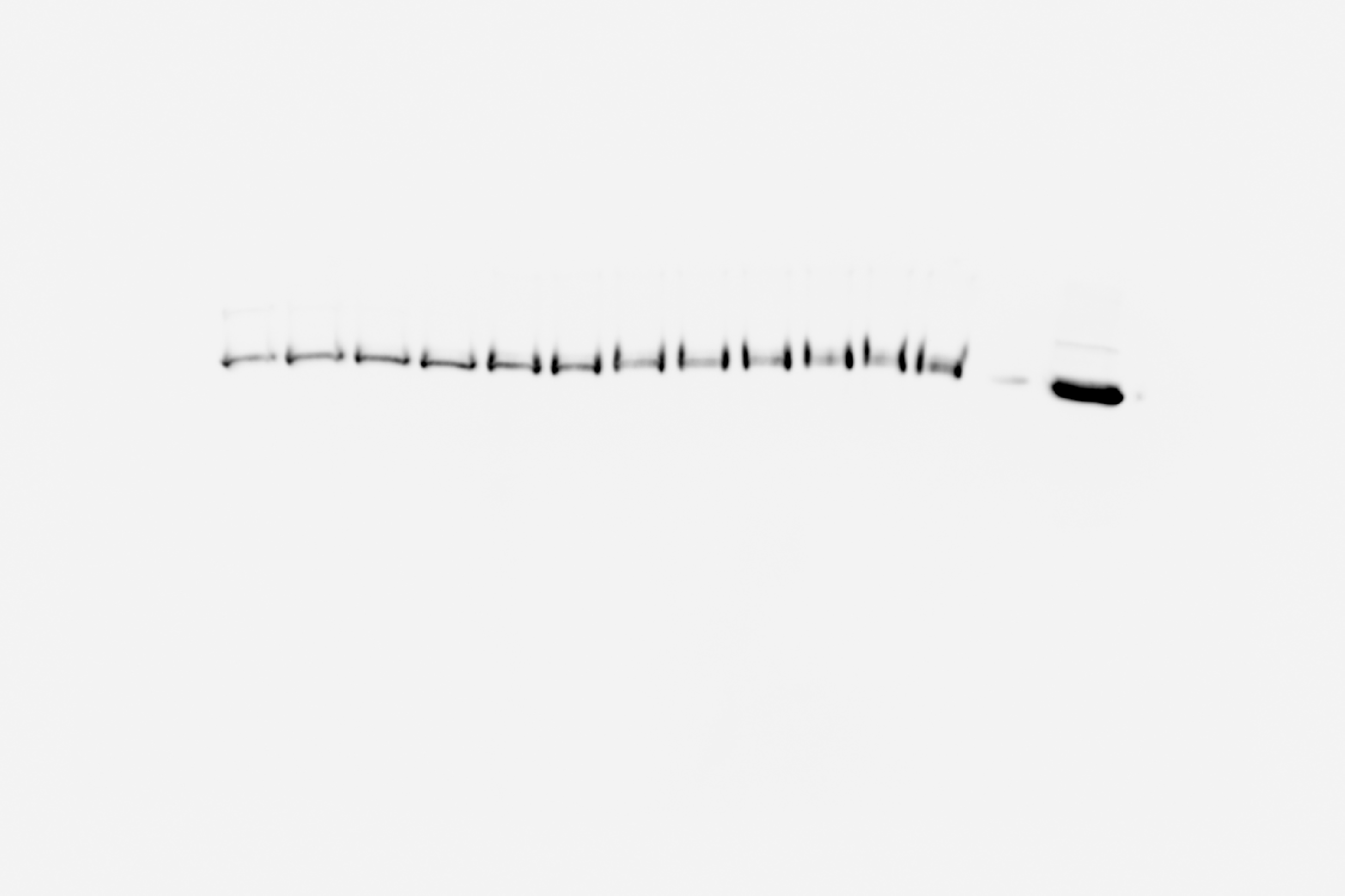

Supplement: Figure 4—source data 3. [file elife-96841-fig4-data3.zip › Figure 4-source data 3/Figure 4E - DVL1-CFRDVL2.tif]

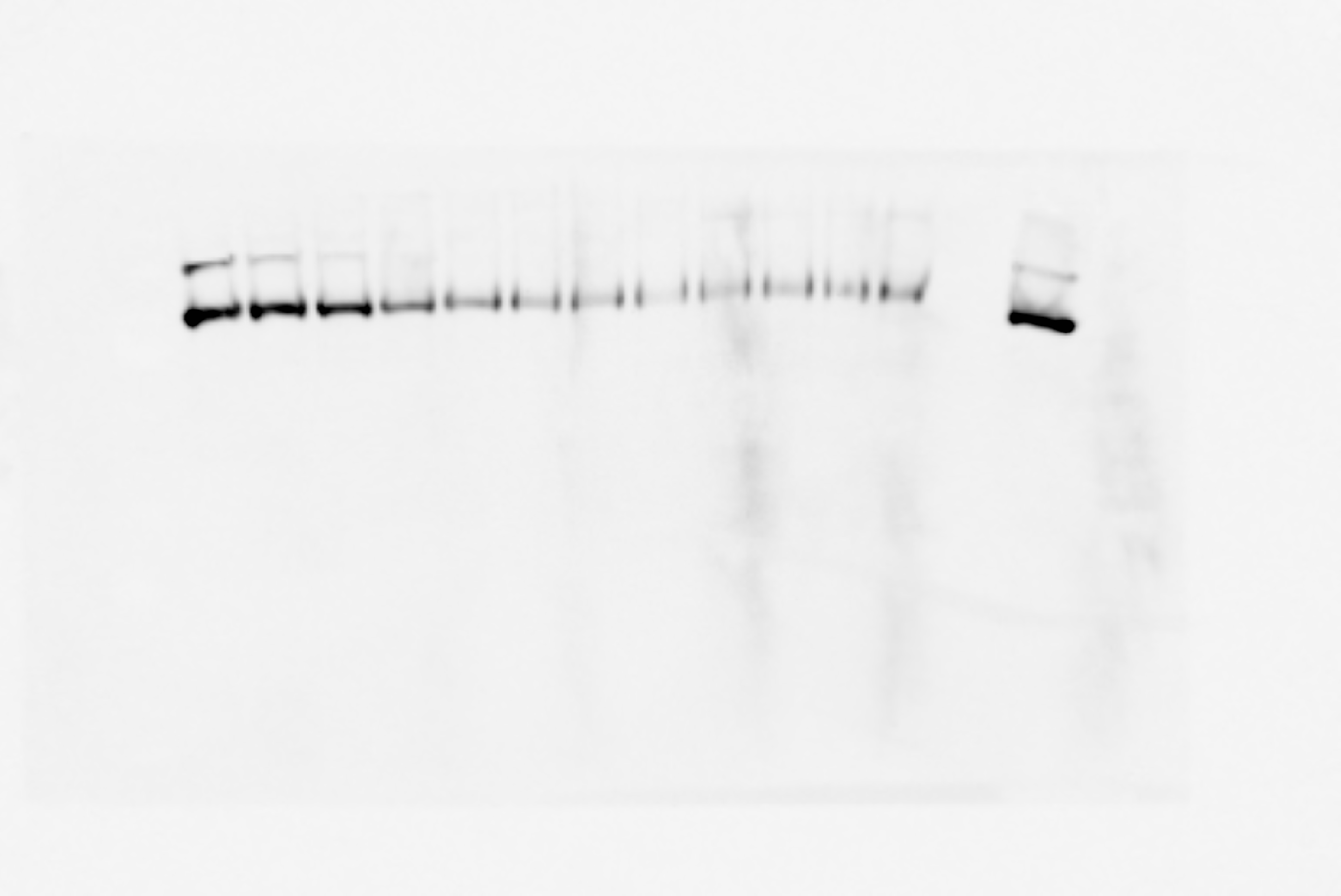

Supplement: Figure 4—source data 3. [file elife-96841-fig4-data3.zip › Figure 4-source data 3/Figure 4E - DVL1.tif]

To Panel I

unedited

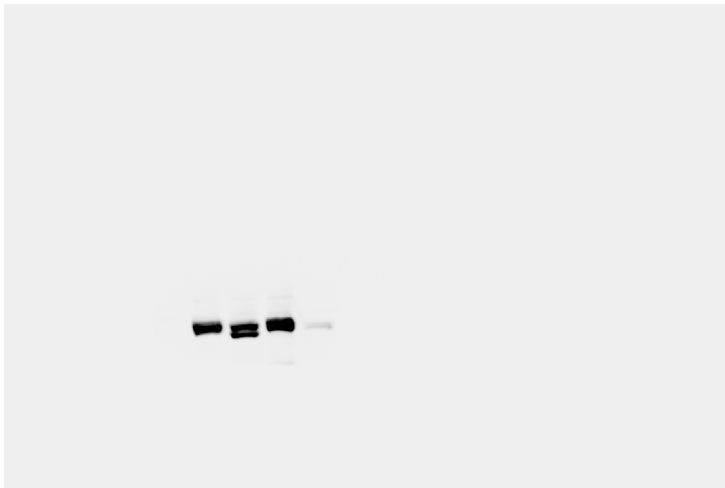

labelled

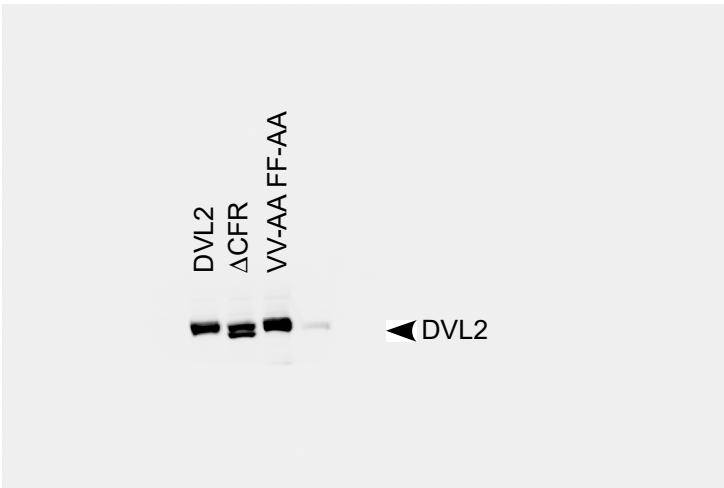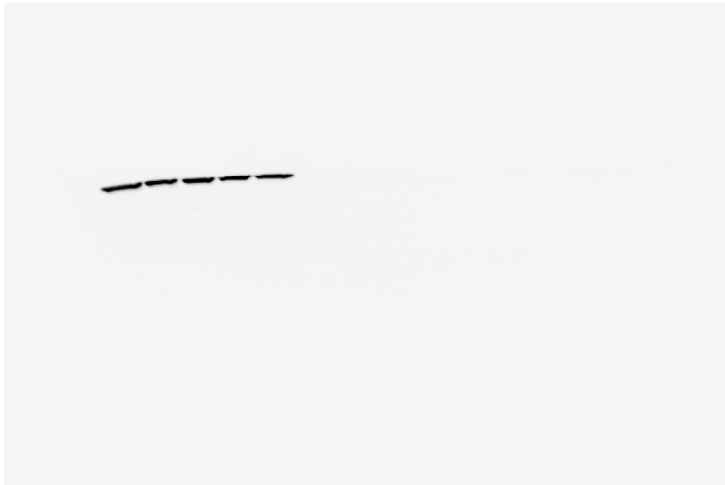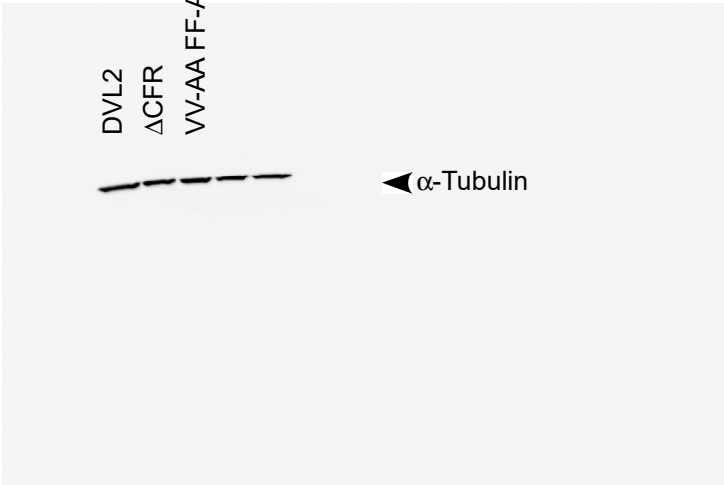

Supplement: Figure 5—figure supplement 1—source data 2. [file elife-96841-fig5-figsupp1-data2.zip › Figure 5-figure supplement 1-source data 2/Figure 5-Figure Supplement 1I.pdf]

To Panel J

unedited

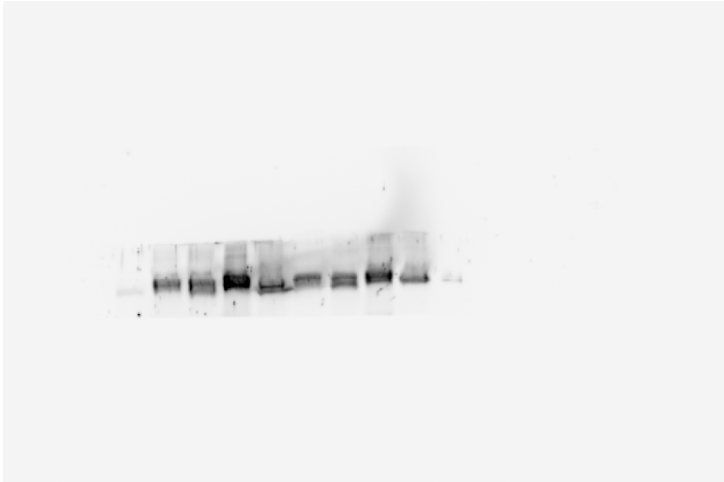

labelled

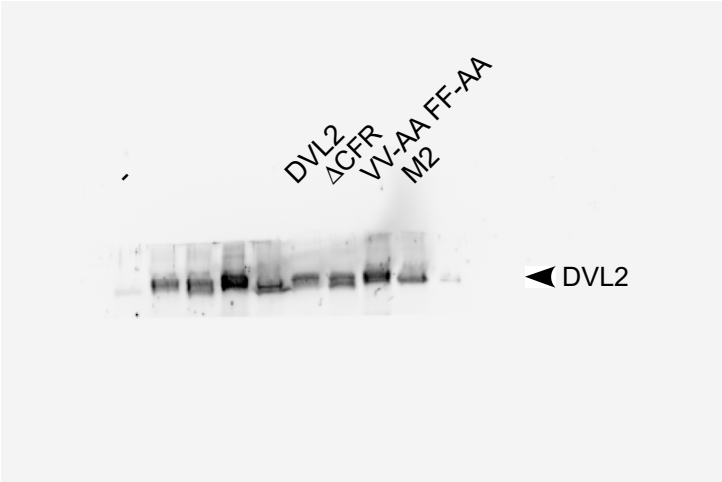

To Panel K

unedited

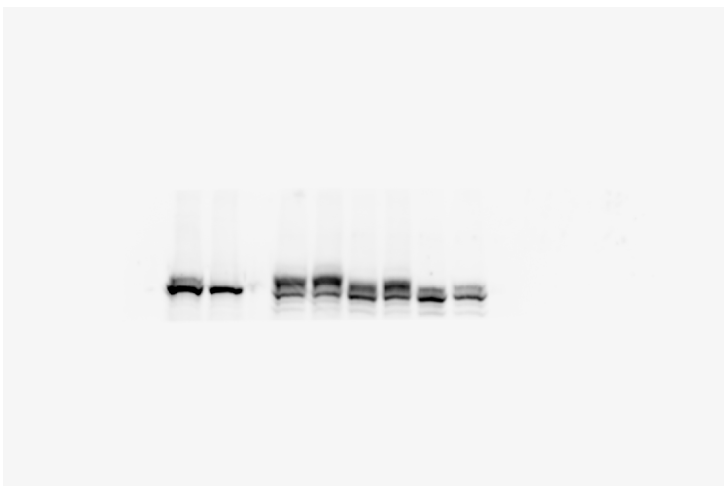

labelled

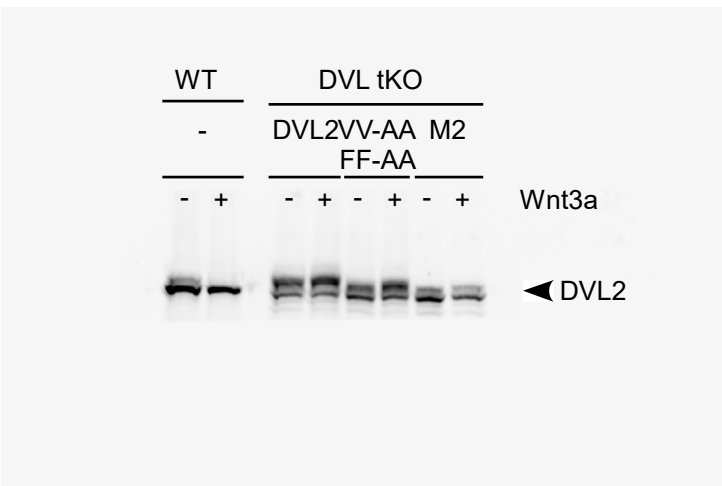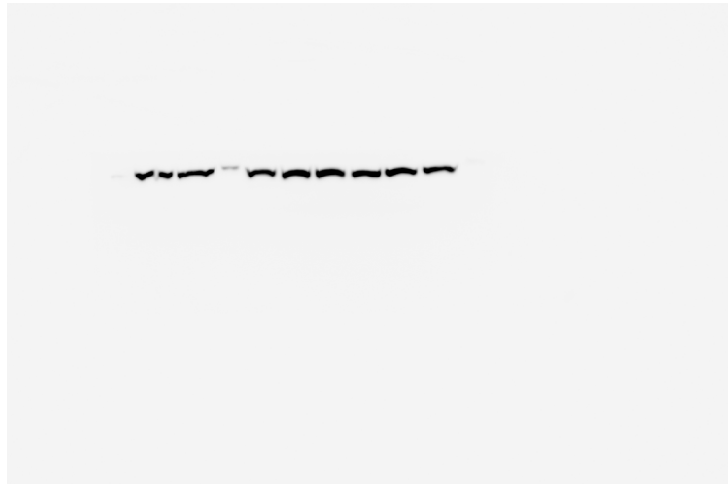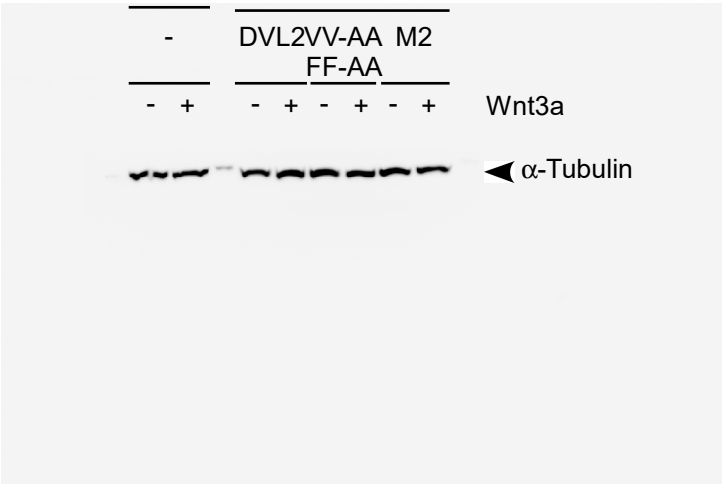

Supplement: Figure 5—figure supplement 1—source data 2. [file elife-96841-fig5-figsupp1-data2.zip › Figure 5-figure supplement 1-source data 2/Figure 5-Figure Supplement 1J and K.pdf]

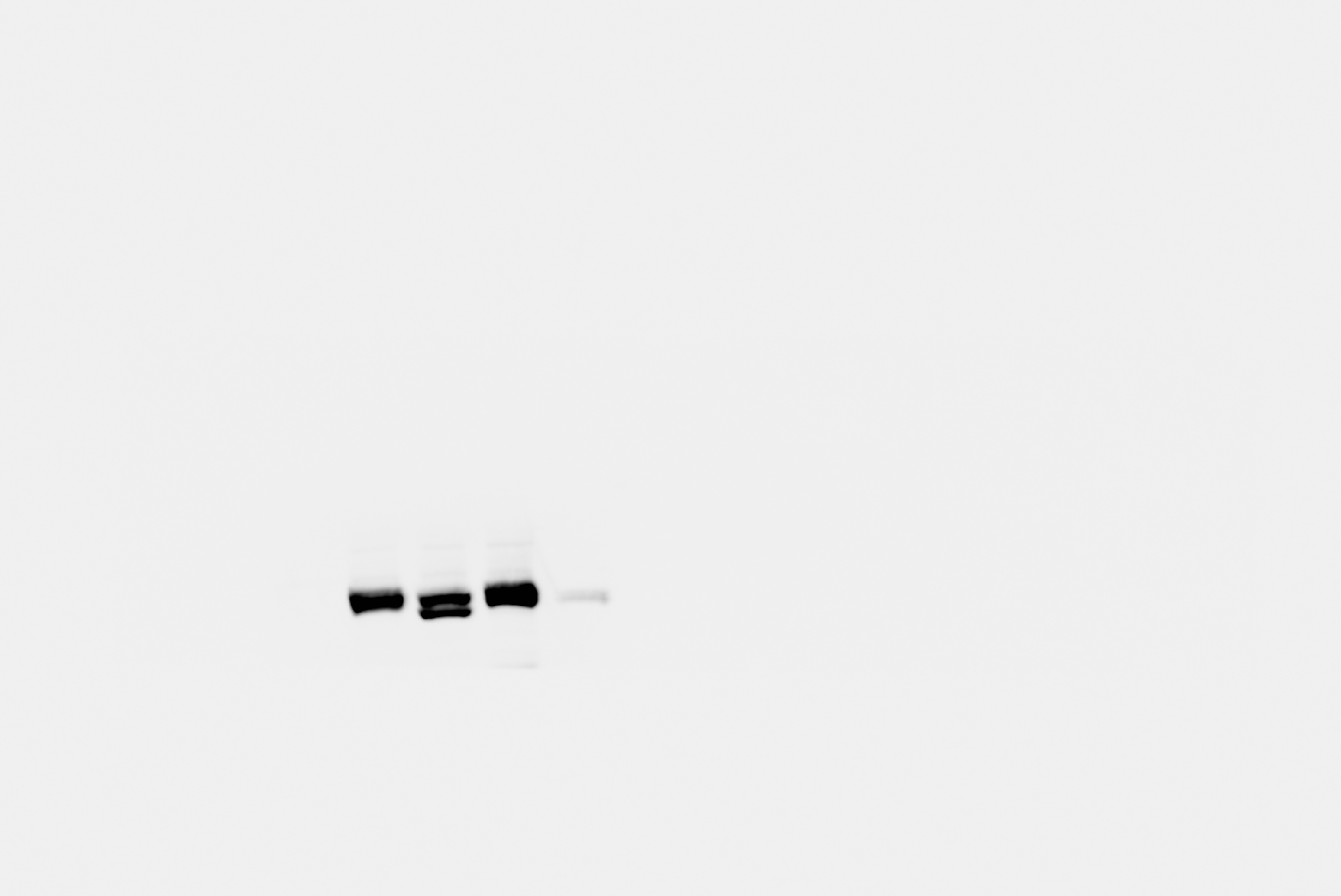

Supplement: Figure 5—figure supplement 1—source data 3. [file elife-96841-fig5-figsupp1-data3.zip › Figure 5-figure supplement 1-source data 3/Figure 5-figure supplement 1I - DVL2.tif]

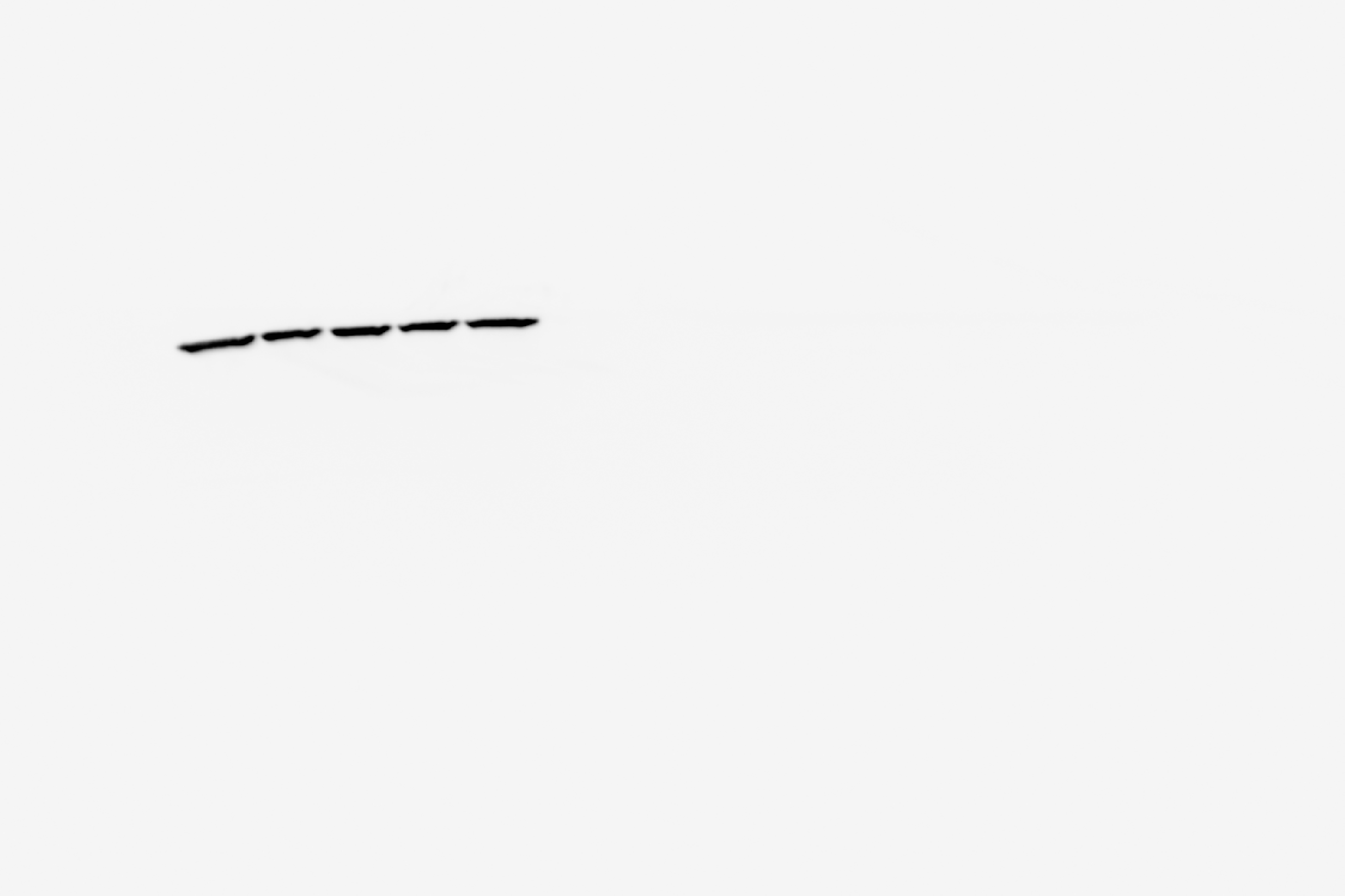

Supplement: Figure 5—figure supplement 1—source data 3. [file elife-96841-fig5-figsupp1-data3.zip › Figure 5-figure supplement 1-source data 3/Figure 5-figure supplement 1I - Tubulin.tif]

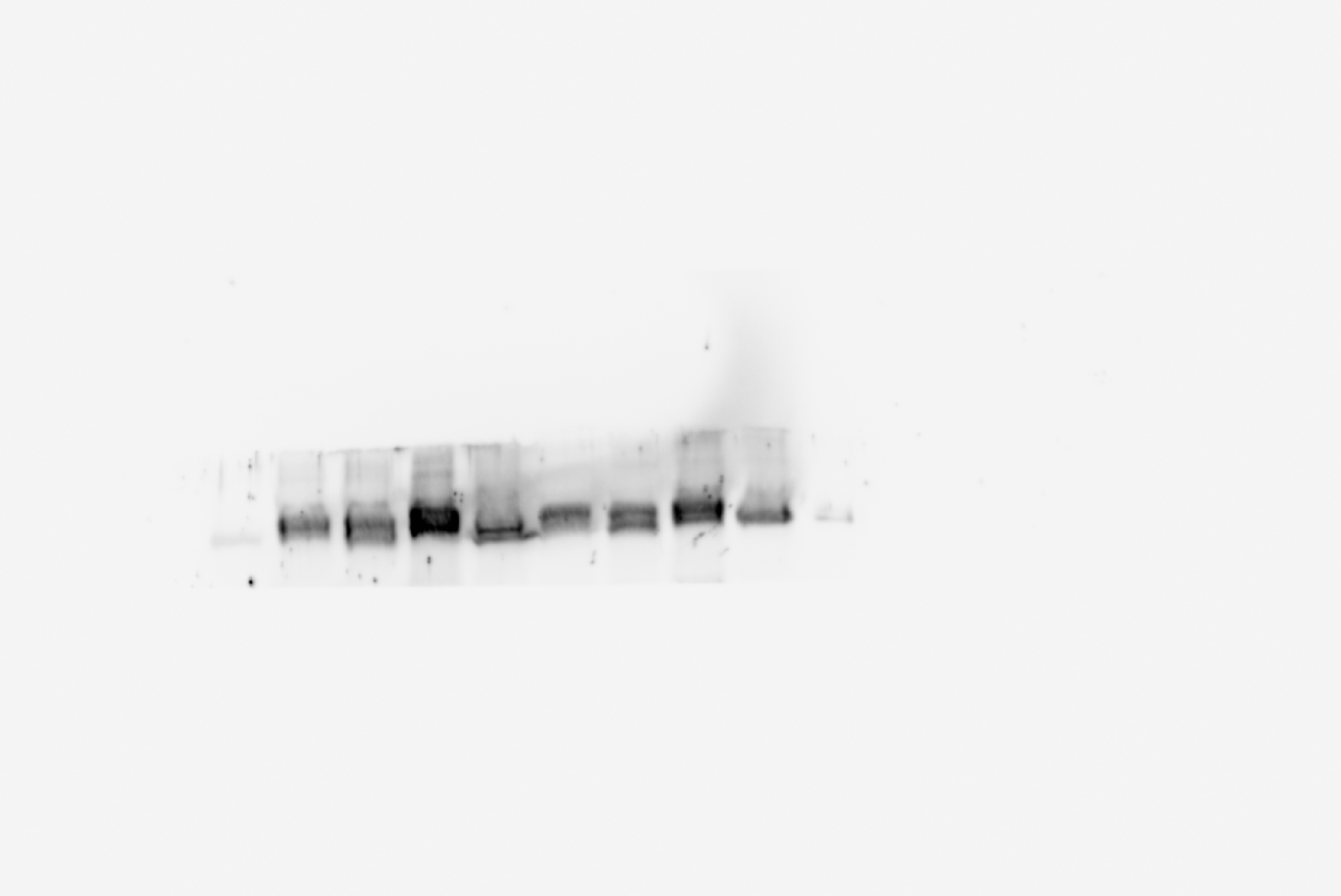

Supplement: Figure 5—figure supplement 1—source data 3. [file elife-96841-fig5-figsupp1-data3.zip › Figure 5-figure supplement 1-source data 3/Figure 5-figure supplement 1J - DVL2.tif]

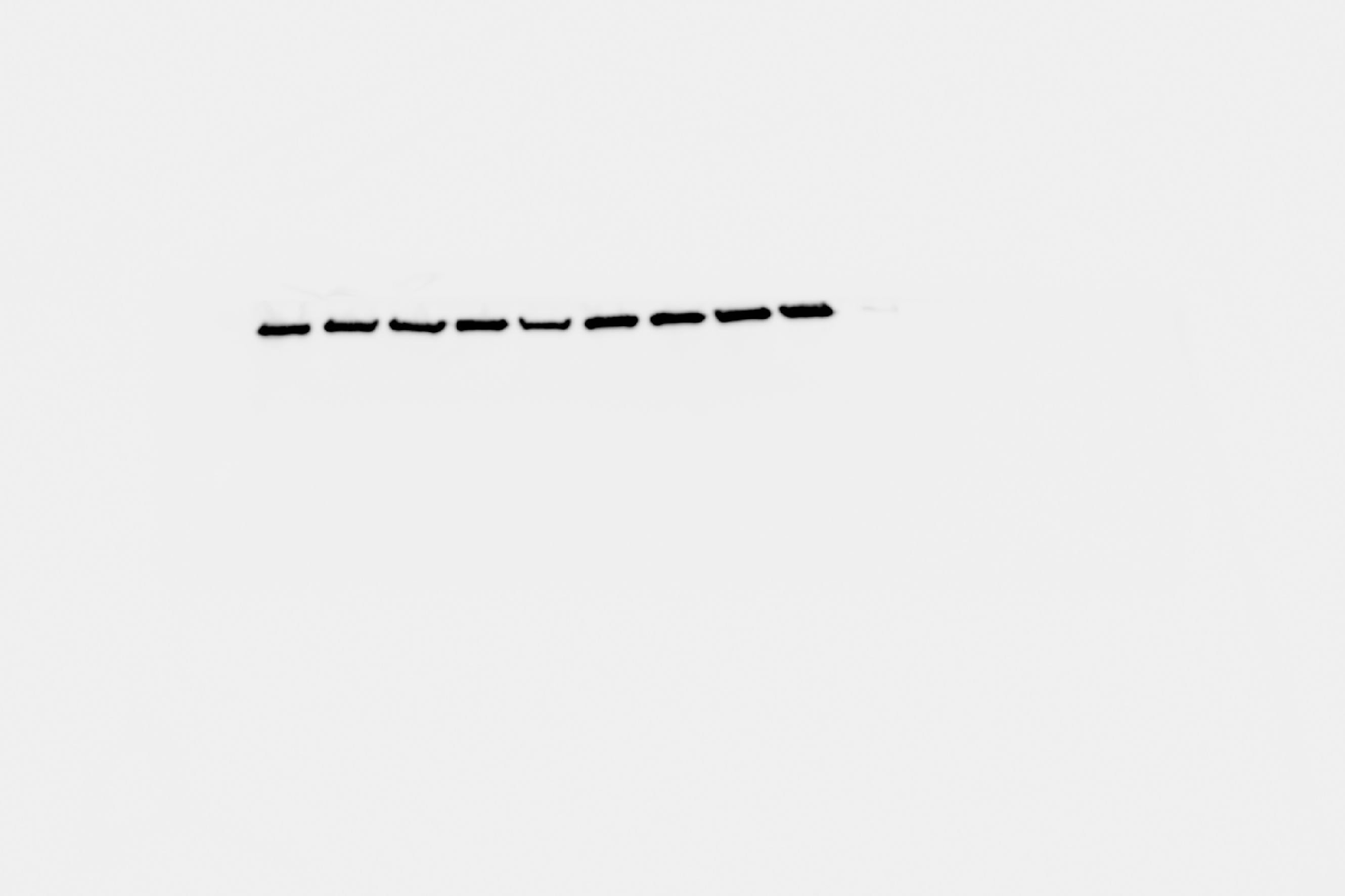

Supplement: Figure 5—figure supplement 1—source data 3. [file elife-96841-fig5-figsupp1-data3.zip › Figure 5-figure supplement 1-source data 3/Figure 5-figure supplement 1J - Tubulin.tif]

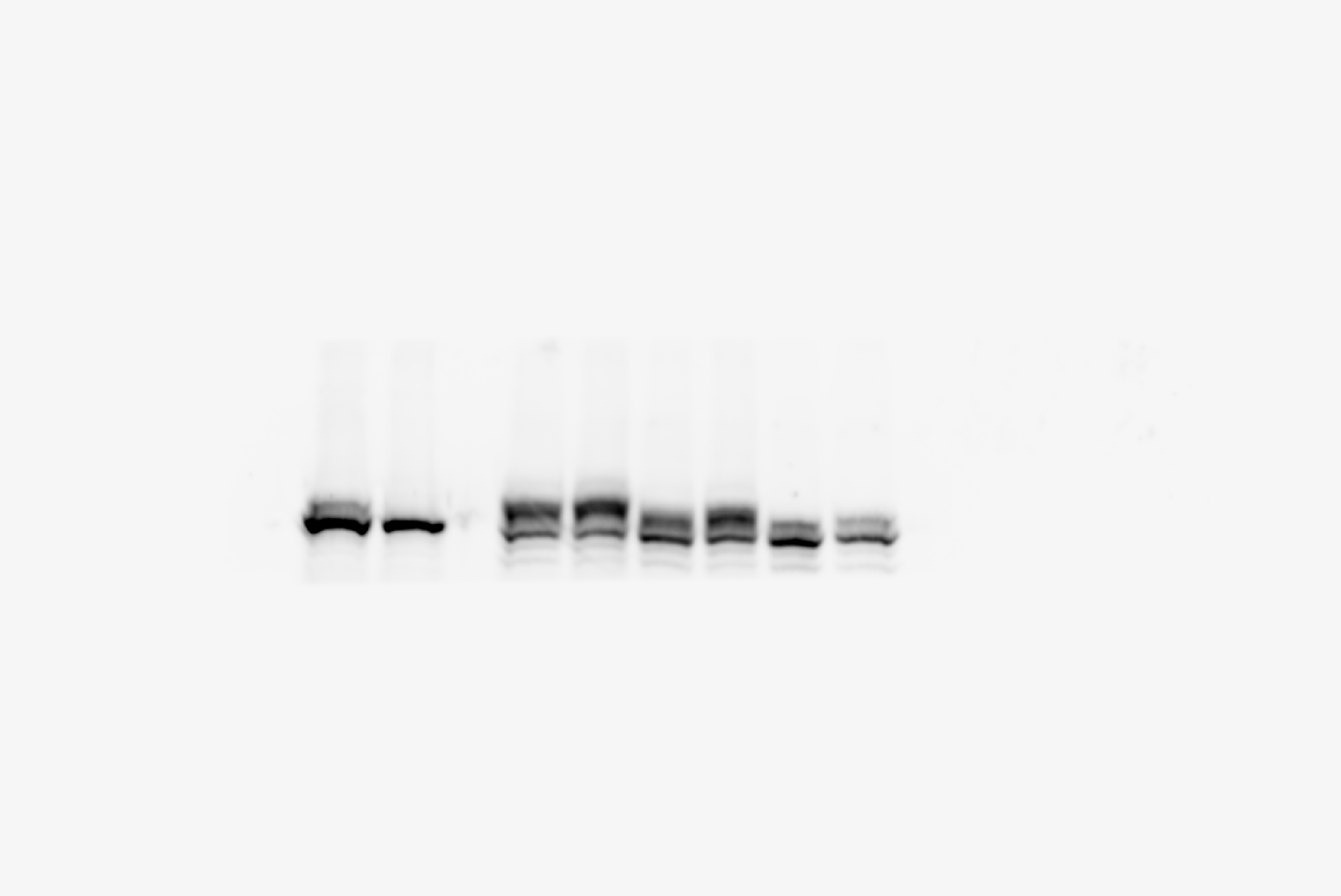

Supplement: Figure 5—figure supplement 1—source data 3. [file elife-96841-fig5-figsupp1-data3.zip › Figure 5-figure supplement 1-source data 3/Figure 5-figure supplement 1K - DVL2.tif]

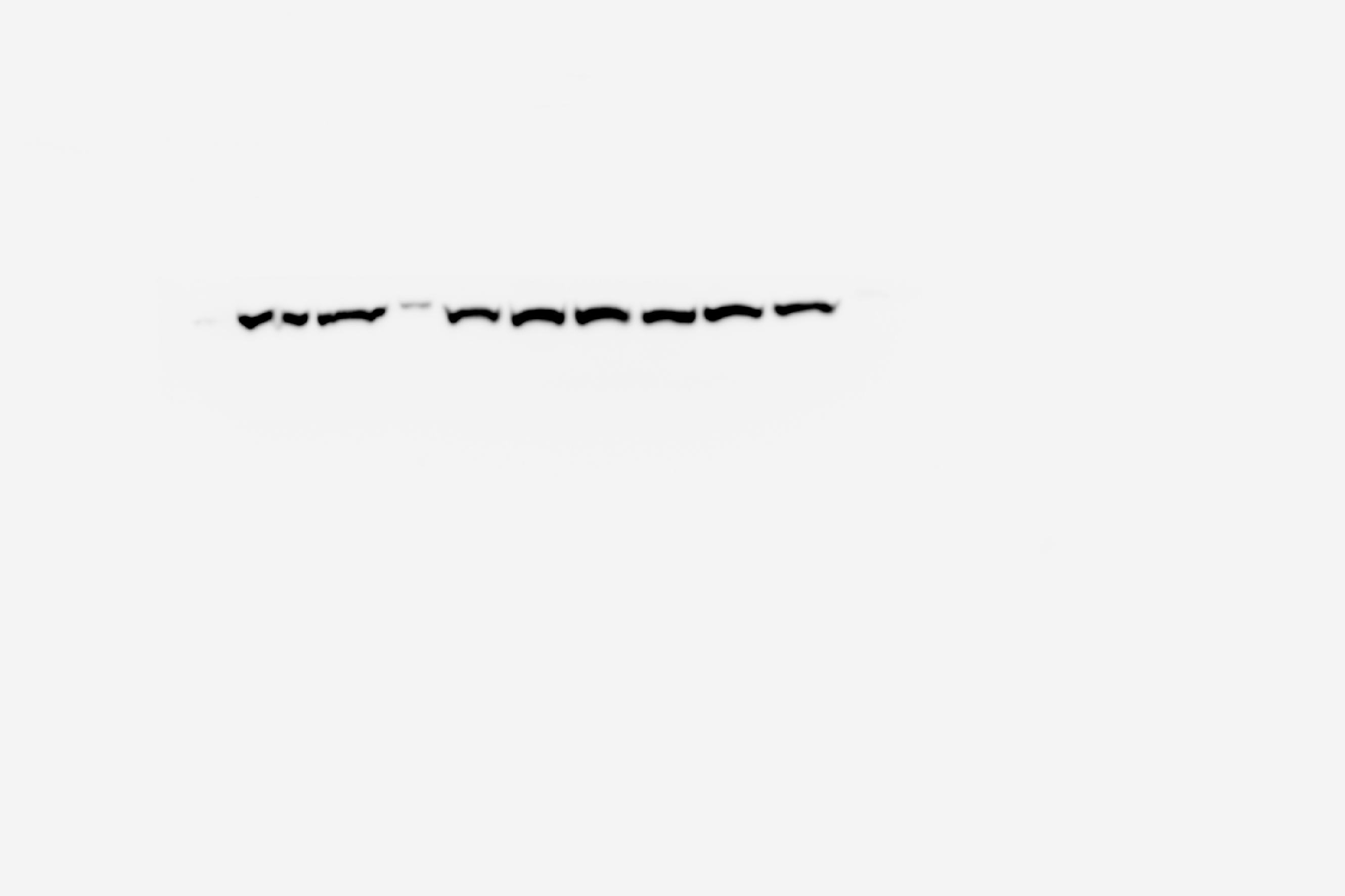

Supplement: Figure 5—figure supplement 1—source data 3. [file elife-96841-fig5-figsupp1-data3.zip › Figure 5-figure supplement 1-source data 3/Figure 5-figure supplement 1K - Tubulin.tif]

To Panel A

unedited

labelled

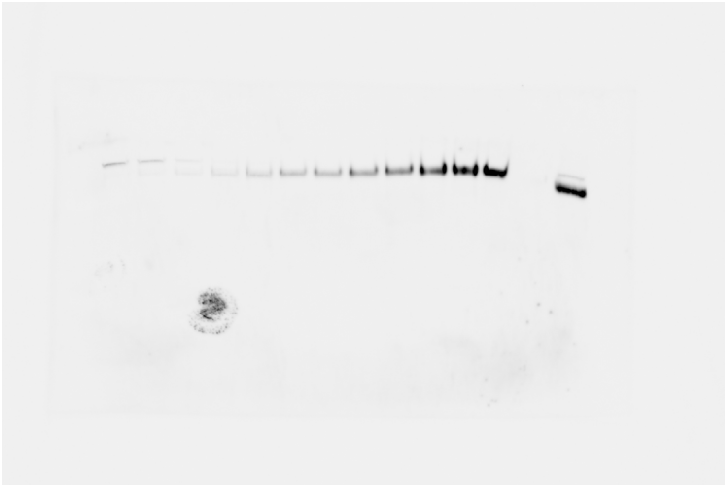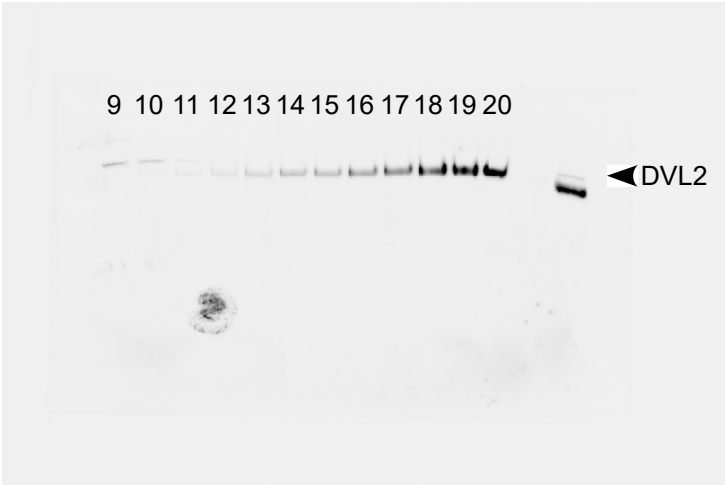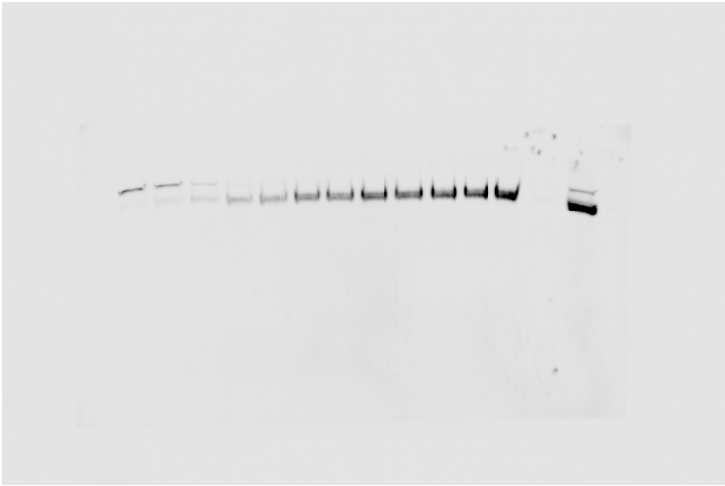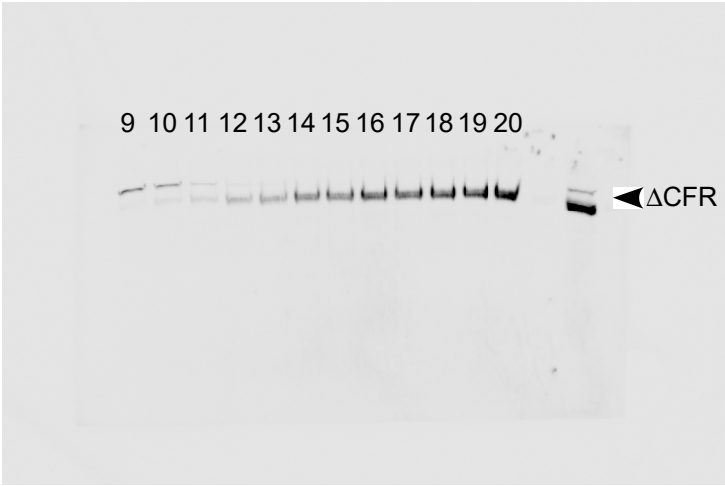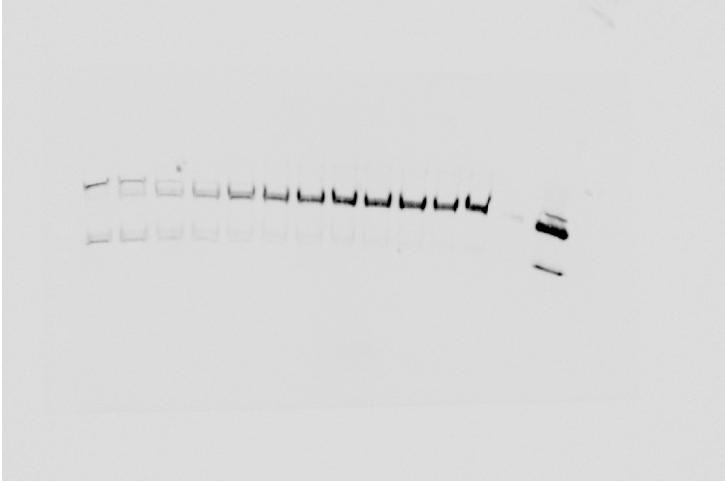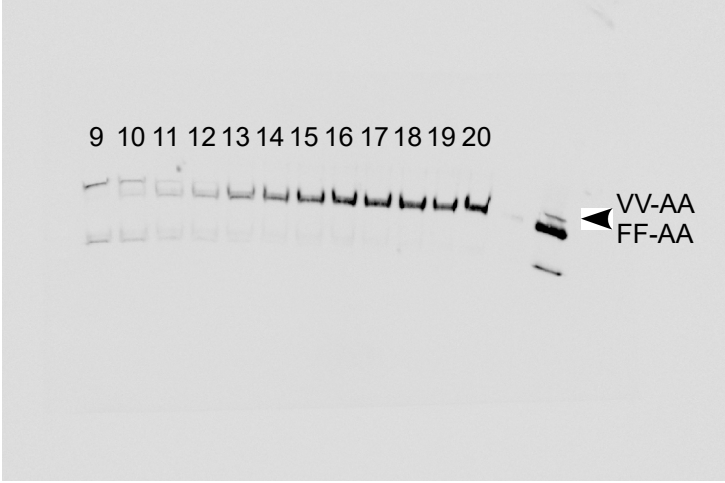

Supplement: Figure 7—source data 2. [file elife-96841-fig7-data2.zip › Figure 7-source data 2/Figure 7A.pdf]

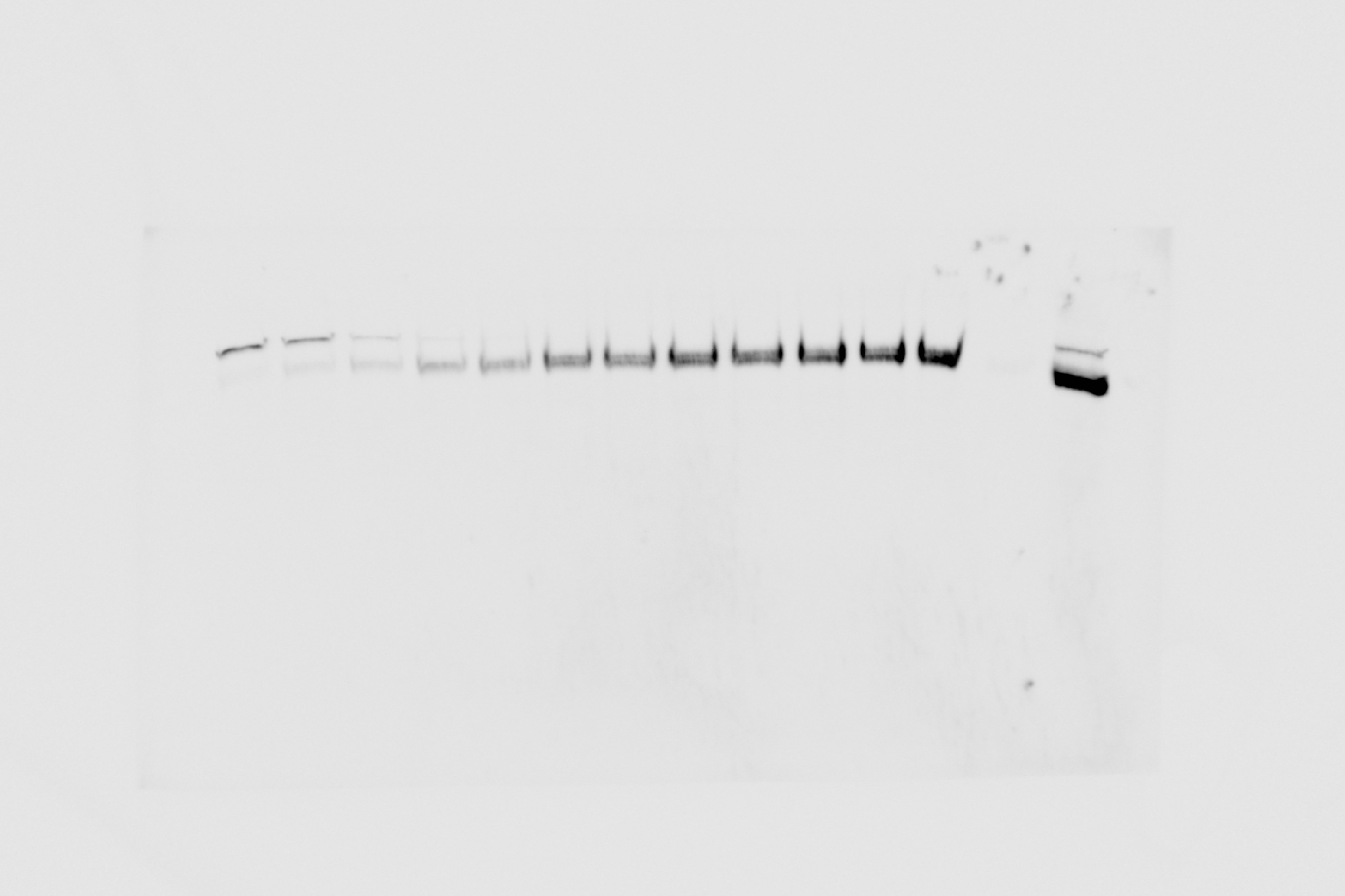

Supplement: Figure 7—source data 3. [file elife-96841-fig7-data3.zip › Figure 7-source data 3/Figure 7A - deltaCFR.tif]

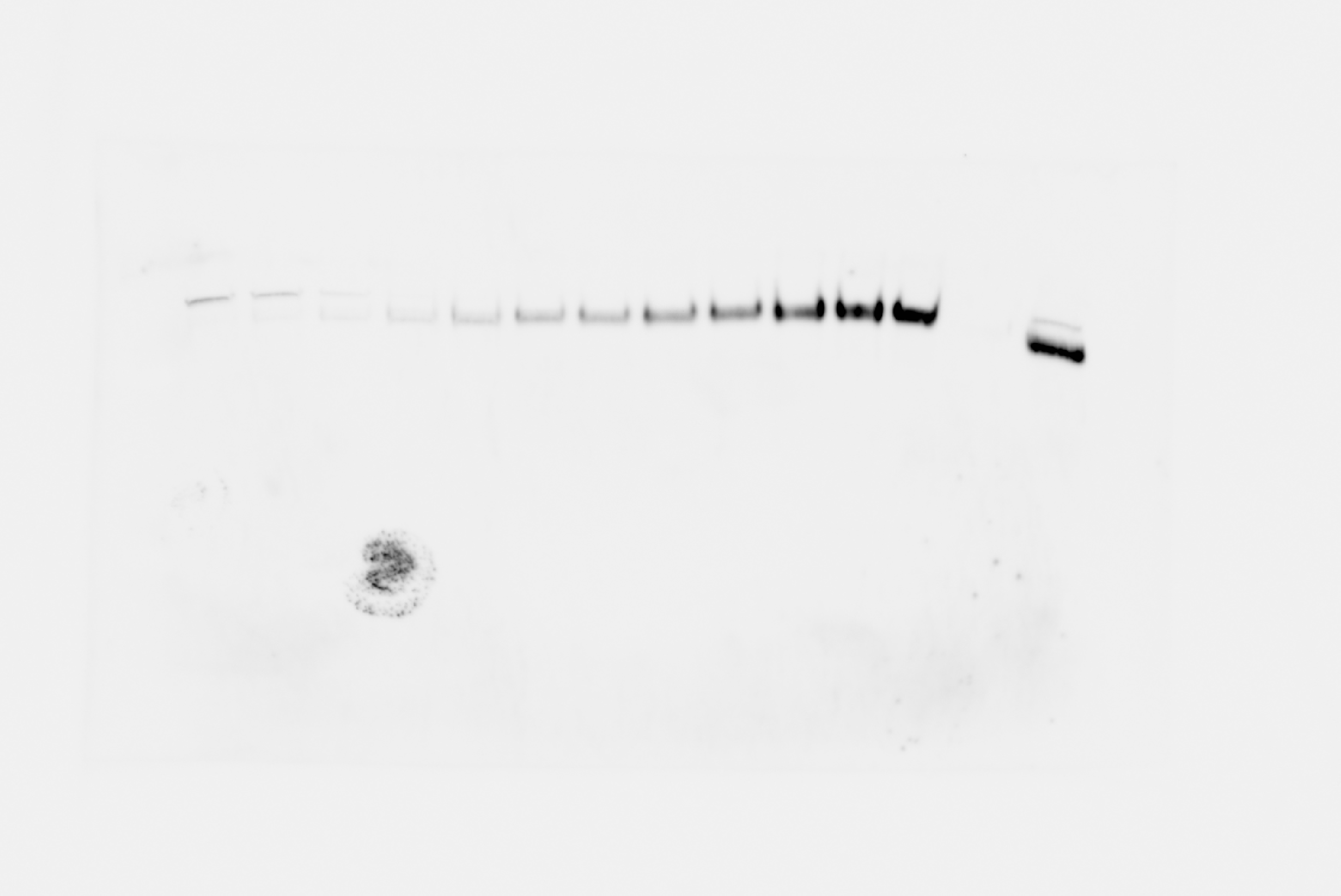

Supplement: Figure 7—source data 3. [file elife-96841-fig7-data3.zip › Figure 7-source data 3/Figure 7A - DVL2.tif]

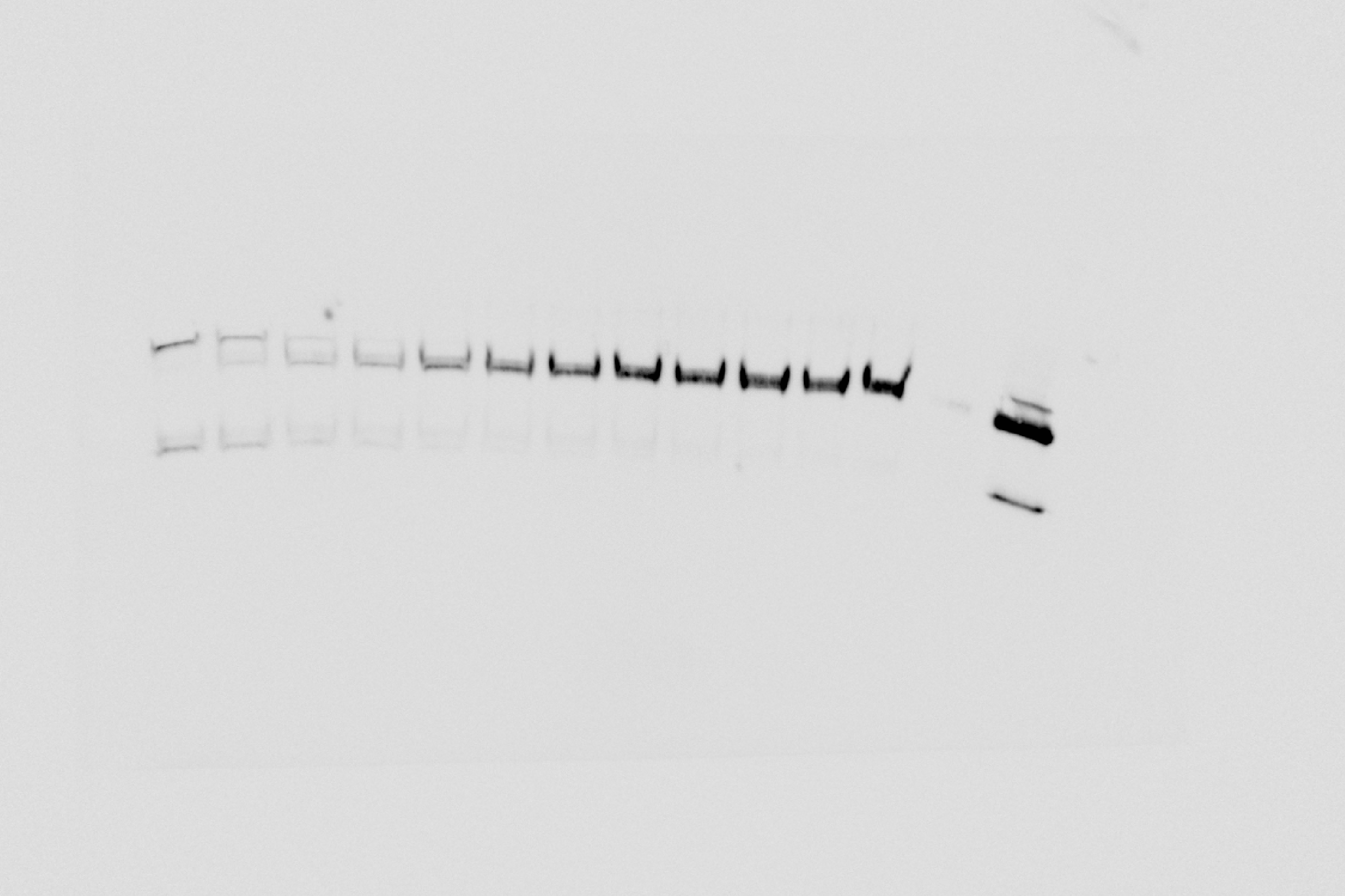

Supplement: Figure 7—source data 3. [file elife-96841-fig7-data3.zip › Figure 7-source data 3/Figure 7A - VV-AA FF-AA.tif]
